# An allosteric conduit facilitates dynamic multisite substrate recognition by the SCF^Cdc4^ ubiquitin ligase

**DOI:** 10.1038/ncomms13943

**Published:** 2017-01-03

**Authors:** Veronika Csizmok, Stephen Orlicky, Jing Cheng, Jianhui Song, Alaji Bah, Neda Delgoshaie, Hong Lin, Tanja Mittag, Frank Sicheri, Hue Sun Chan, Mike Tyers, Julie D. Forman-Kay

**Affiliations:** 1 Molecular Structure & Function, The Hospital for Sick Children, Toronto, Ontario, Canada M5G 0A4; 2 Lunenfeld-Tanenbaum Research Institute, Mount Sinai Hospital, Toronto, Ontario, Canada M5G 1X5; 3 Institute for Research in Immunology and Cancer, Université de Montréal, Montréal, Quebec, Canada H3C 3J7; 4 Department of Biochemistry, University of Toronto, Toronto, Ontario, Canada M5S 1A8; 5 Department of Molecular Genetics, University of Toronto, Toronto, Ontario, Canada M5S 1A8; 6 School of Polymer Science and Engineering, Qingdao University of Science and Technology, Shandong 266042, China

## Abstract

The ubiquitin ligase SCF^Cdc4^ mediates phosphorylation-dependent elimination of numerous substrates by binding one or more Cdc4 phosphodegrons (CPDs). Methyl-based NMR analysis of the Cdc4 WD40 domain demonstrates that Cyclin E, Sic1 and Ash1 degrons have variable effects on the primary Cdc4^WD40^ binding pocket. Unexpectedly, a Sic1-derived multi-CPD substrate (pSic1) perturbs methyls around a previously documented allosteric binding site for the chemical inhibitor SCF-I2. NMR cross-saturation experiments confirm direct contact between pSic1 and the allosteric pocket. Phosphopeptide affinity measurements reveal negative allosteric communication between the primary CPD and allosteric pockets. Mathematical modelling indicates that the allosteric pocket may enhance ultrasensitivity by tethering pSic1 to Cdc4. These results suggest negative allosteric interaction between two distinct binding pockets on the Cdc4^WD40^ domain may facilitate dynamic exchange of multiple CPD sites to confer ultrasensitive dependence on substrate phosphorylation.

## Abstract (web summary)

The WD40 domain of the SCF^Cdc4^ ubiquitin ligase targets substrates via multiple phosphorylated degron motifs. The authors define a second degron-binding WD40 pocket that imparts a negative allosteric effect on binding to the primary pocket, and thereby enables the dynamic exchange of bound degrons.

WD40 domains, and the related Kelch β-propeller domains, mediate protein interactions in many contexts and together form the largest domain family in the human genome^1^. WD40 domain proteins are a predominant class of substrate receptor in the ubiquitin-proteasome system, found in F-box proteins and other subunits of the cullin-RING (CRL) ubiquitin ligases^2^,^3^,^4^. The WD40 domain of the yeast F-box protein Cdc4 (Cdc4^WD40^) recognizes substrates via phosphorylated motifs termed Cdc4 phosphodegrons (CPDs). One of the best characterized Cdc4 substrates is the cyclin dependent kinase inhibitor Sic1, which is targeted for ubiquitination and degradation in late G1 phase upon its phosphorylation by Cln-Cdc28 kinase activity^5^,^6^. Sic1 is phosphorylated at 9 low-affinity CPD sites (*K*_d_ ∼10-100 μM) that all bear mismatches to the optimal CPD sequence (I/L-I/L/P-pT-P-<KR>4, where<> indicates a disfavoured residue)^7^. Phosphorylation on between 4 and 6 low-affinity CPD sites generates a high-affinity Sic1-Cdc4 interaction (*K*_d_∼1 μM)^7^,^8^. This requirement for multisite phosphorylation sets a threshold that converts a graded increase in Cln-Cdc28 activity into switch-like elimination of Sic1^6^,^8^. Responses that transform graded inputs into sharply thresholded outputs are referred to as ‘ultrasensitive'^9^,^10^, and often result from multisite phosphorylation^11^,^12^.

Nuclear magnetic resonance (NMR) analysis suggests that multiple Sic1 low-affinity CPD sites interact with Cdc4 in a dynamic equilibrium to generate an overall high-affinity interaction^13^,^14^. The structure of a high-affinity singly phosphorylated CPD bound to Cdc4 revealed that the P0 phosphorylated residue occupies a deep arginine-rich pSer/Thr binding pocket in the WD40 domain, and that adjacent sub-pockets confer sequence selectivity at P−2, P−1, P+1 and P+2 CPD positions^7^. A distal basic surface in Cdc4 and its human orthologue Fbw7 can also simultaneously engage a second phosphorylated residue at the P+4 position to increase affinity^15^,^16^,^17^, or conversely antagonize the interaction of CPDs with C-terminal basic residues^7^. However, this secondary phosphate-binding site is dispensable for Sic1 degradation *in vivo*^8^. Dimerization of the SCF^Fbw7^ complex also contributes to the recognition of some multisite phosphorylated substrates^18^, and catalytic efficiency of the SCF^Cdc4^ complex^19^. Cln- and Clb-CDK may act in a processive multi-phosphorylation cascade on Sic1 to facilitate Cdc4 recognition through generation of appropriate CPD sites^16^. Recognition of the Ash1 transcriptional repressor by Cdc4 depends on three closely-spaced CPD sites that form a redundant diphosphodegron^20^. Other substrates, including Far1, Gcn4, Cln2 and Cln3 for Cdc4 in yeast and cyclin E, c-Myc and Mcl1 for Fbw7 in humans, also may depend on multisite phosphorylation^2^,^21^,^22^. These various interaction modes illustrate the tunability of the Cdc4/Fbw7 system for substrate elimination.

The phenomenon of allostery, whereby ligand binding at one site alters interactions at a physically distant site, allows for complex control of protein function^23^,^24^,^25^. A biplanar dicarboxylic acid compound called SCF-I2 allosterically inhibits Cdc4^WD40^-substrate interactions by binding to a site 25 Å away from the main CPD pocket^26^. SCF-I2 elicits this allosteric effect through structural changes that occlude the P−2 region of the CPD pocket. Enigmatically, SCF-I2 is 10-fold less potent towards full-length Sic1 compared to a singly phosphorylated high-affinity CPD of similar affinity for Cdc4. The SCF-I2 site and associated allosteric infrastructure appear to be conserved through evolution^27^.

The large size of the Cdc4 complex previously precluded determination of substrate interaction effects on the WD40 domain. Here we investigate substrate-Cdc4 interactions by NMR analysis of assigned methyl groups in the isolated Cdc4^WD40^ domain. Unexpectedly, we find that the CPDs of an extended phosphorylated Sic1 substrate directly contact the SCF-I2 allosteric site. On the basis of this data and the inhibitory effect of SCF-I2 on the CPD pocket, we predicted that extended multisite CPDs might engage the WD40 domain through both pockets in a mutually antagonistic manner, and then confirmed this hypothesis using fluorescence-binding data. Consistent with this negative allosteric effect, NMR analysis reveals smaller chemical shifts in the P−2 sub-pocket for extended multisite CPDs compared with short CPD-containing peptides. Mathematical modelling suggests that interactions at the primary and allosteric pockets may enhance the ultrasensitive dependence of the Sic1-Cdc4 interaction on Sic1 phosphorylation. This negative allosteric binding mechanism helps explain the conundrum of how multiple low-affinity sites can generate a rapidly exchangeable yet high-affinity Sic1-Cdc4 interaction.

## Results

### Characterization of isolated Cdc4^WD40^

We determined the effects of substrate binding on the isolated WD40 domain of Cdc4 (Cdc4^WD40^), which is more amenable to NMR due to its size (40 kDa) than the minimal Cdc4-Skp1 complex (∼70 kDa). The N-terminal targeting region of Sic1 (residues 1-90, called Sic1^1-90^), which contains 7 of the 9 Sic1 CPD sites and is sufficient for Cdc4 binding^28^, was phosphorylated to completion by Cln2-Cdc28-Cks1 kinase (called pSic1). We used isothermal titration calorimetry (ITC) to determine the affinity of Cdc4^WD40^ for pSic1 and SCF-I2 (Supplementary Fig. 1a–c). The *K*_d_ value for pSic1-Cdc4^WD40^ was 0.8±0.12 μM, similar to the 0.6 μM value determined for pSic1-Cdc4-Skp1 by intrinsic tryptophan (Trp) fluorescence^13^. The *K*_d_ value for SCF-I2 binding Cdc4^WD40^ was 0.6±0.02 μM, concordant with the ∼2 μM *K*_i_ value determined for SCF-I2-Cdc4-Skp1 by a fluorescence polarization competition assay^26^.

**Figure 1:**
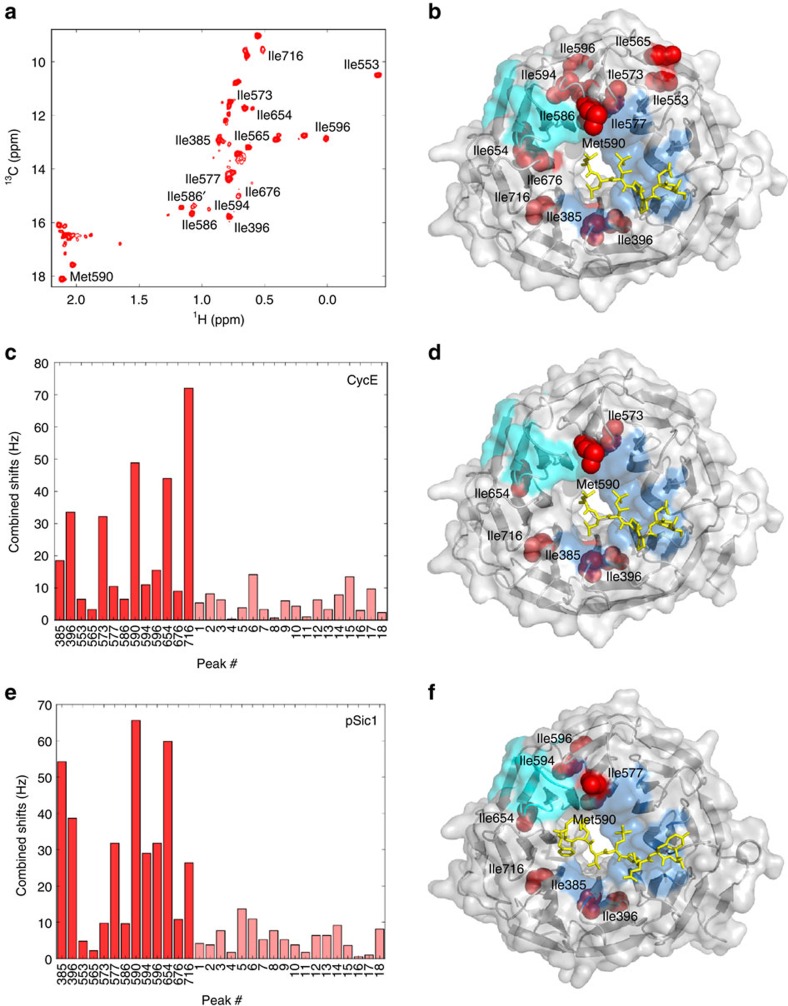
NMR titration of IM-labelled Cdc4^WD40^ with CycE and pSic1. (**a**) ^1^H–^13^C TROSY HMQC spectrum of Cdc4^WD40^ with assigned Ile and Met resonances. (**b**) Surface and ribbon representation of Cdc4^WD40^ with CycE peptide (PDB ID 1NEX) in yellow, CPD-binding pocket^7^ in blue, SCF-I2 binding pocket^26^ in cyan and assigned residues in red spheres. (**c**) and (**e**) Combined ^1^H and ^13^C chemical shift perturbations of Cdc4^WD40^ upon (**c**) CycE or (**e**) pSic1 binding. Assigned Ile and Met residues are in red, labelled with residue number; unassigned residues in pink. (**d**) and (**f**) Surface and ribbon representation of Cdc4^WD40^ with (**d**) CycE peptide (1NEX) or (**f**) Sic1 peptide (3V7D). Colouring as for (**b**) except only residues showing significant chemical shift changes are highlighted in red spheres.

NMR resonances for Cdc4^WD40^ were highly broadened, with millisecond*–*microsecond timescale motion likely playing a significant role. Although addition of a CycE phosphopeptide or SCF-I2 sharpened resonances (Supplementary Fig. 1d,e), backbone assignment was not possible. However, as Cdc4^WD40^ methyl resonances were sharper due to the favourable properties of methyls^29^,^30^, we analysed Ile-δ1-[^13^CH_3_] and Met-ɛ-[^13^CH_3_] (IM)-labelled samples (Fig. 1a). Cdc4^WD40^ contains 24 Ile and 5 Met residues distributed throughout the WD40 domain. Three Ile (Ile573, Ile716 and Ile385) and 1 Met (Met590) are adjacent to the arginine-rich CPD binding pocket on Cdc4 and another 9 Ile residues lie in close proximity to both the CPD and the allosteric pockets. Point mutations of 15 Ile to Val or Met to Val enabled assignment of 12 Ile and 1 Met methyls in Cdc4^WD40^ (Fig. 1b).

### Perturbation of Cdc4^WD40^ methyl resonances

Unlabelled CycE phosphopeptide, pSic1 or various phosphopeptides derived from Sic1 and Ash1 were titrated into IM-labelled Cdc4^WD40^ samples and chemical shift perturbations and changes in line shape were observed as a function of ligand concentration. Ligand interactions are expected to broaden NMR spectra due to slowed tumbling, exchange between free and protein/peptide-bound states, and dynamic exchange within the bound state (Supplementary Note 1). Upon titration of CycE phosphopeptide into IM-labelled Cdc4^WD40^, large chemical shift perturbations (>20 Hz combined ^1^H/^13^C shifts, see Methods, Fig. 1c,d, Supplementary Fig. 2a) were observed for Ile396^P^, Ile573^P^, Met590^P^ and Ile716^P^ (where P indicates a residue close to the primary CPD binding pocket) and Ile654 (which lies between the primary CPD and allosteric inhibitor pockets). We also observed a smaller but still significant chemical shift perturbation on Ile385^P^. These NMR results are consistent with engagement of the primary CPD pocket by CycE (Fig. 1d).

Titration of unlabelled pSic1 into IM-labelled Cdc4^WD40^ resulted in large chemical shift perturbations for residues Ile385^P^, Ile396^P^ and Met590^P^ in the primary CPD pocket, as for CycE. Perturbations around the CPD pocket induced by pSic1 matched expectations from X-ray structures of CycE and pSic1 phosphopeptide-Cdc4 complexes^7^,^8^ (Fig. 1d,f). We also observed differences for pSic1 binding compared to CycE, including smaller chemical shift perturbation for Ile716^P^ and no change for Ile573^P^. In contrast to CycE, pSic1 elicited extensive chemical shift perturbations on blade 5 (Ile577^A^, Ile594^A^ and Ile596^A^, where A indicates a residue close to the allosteric inhibitor binding pocket) and blade 6 (Ile654) of Cdc4^WD40^, precisely around the SCF-I2 allosteric binding site (Fig. 1f; Supplementary Fig. 2b).

To deconvolute effects of the multiple CPDs in pSic1, short phosphopeptides (Sic1^10pT2/pT5^, Sic1^9pT45^, Sic1^9pT173^ and Sic1^20pS69/pS76/pS80^) were titrated into IM-labelled Cdc4^WD40^. Each perturbed methyl chemical shifts in the primary pocket (Supplementary Fig. 3), as for CycE. However, only the longer Sic1^20pS69/pS76/pS80^ phosphopeptide perturbed chemical shifts on blade 5 (Ile594^A^, Ile596^A^, Ile577^A^). These data suggested either that in the context of an extended multisite substrate suboptimal CPD sequences affect the SCF-I2 binding pocket through allosteric transmission from the primary CPD pocket or that the CPDs directly contact the SCF-I2 binding pocket.

### The allosteric binding pocket directly engages pSic1

**Figure 2:**
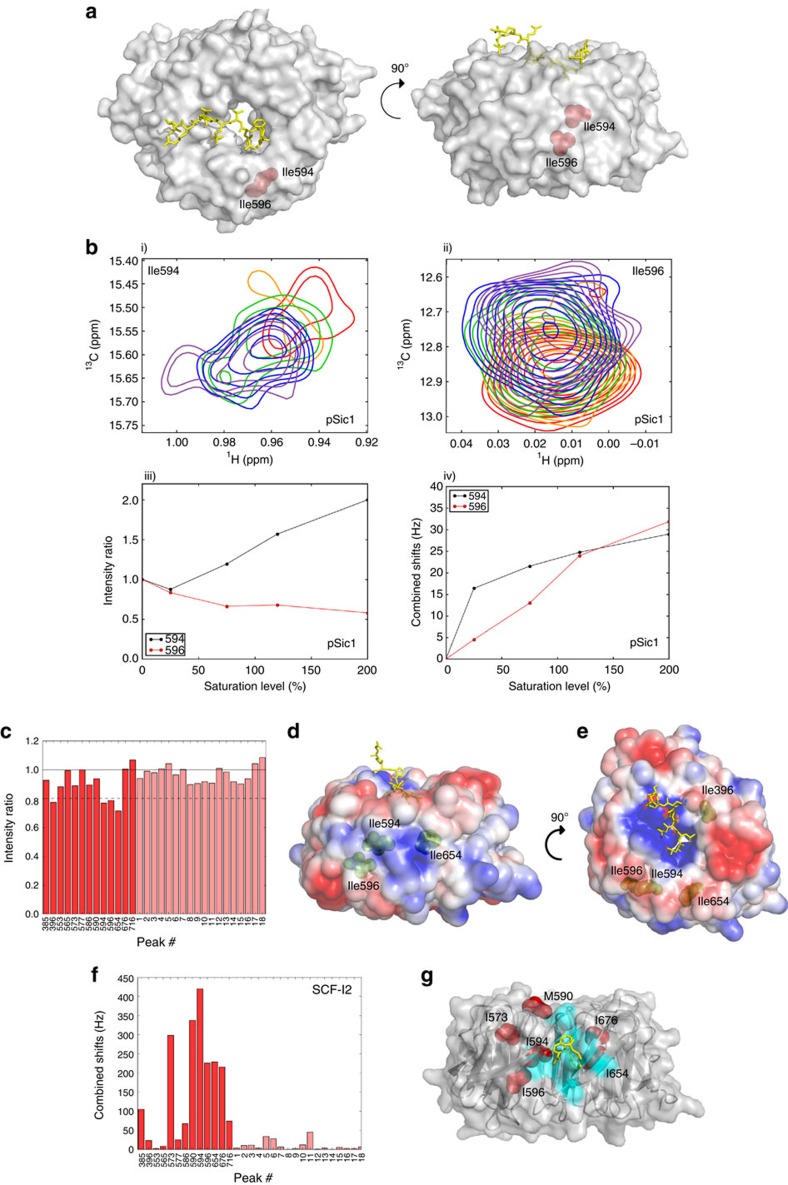
pSic1 and SCF-I2 binding to the allosteric pocket of Cdc4^WD40^. (**a**) Surface representation of Cdc4^WD40^ with Sic1 peptide in yellow and residues in the allosteric pocket in red spheres. (**b**) ^1^H–^13^C TROSY HMQC spectra of residues (i) Ile594 and (ii) Ile596 in the absence (red) and presence of increasing amounts 50% (orange), 75% (green), 120% (blue), 200% (purple) of pSic1, (iii) intensity ratios of the peaks corresponding to the unbound states and (iv) chemical shifts of the peaks corresponding to the bound states at different saturation levels. (**c**) Ratio of signal intensities originating from Ile and Met methyl groups with and without irradiation in the cross-saturation experiments. Intensity ratio of 1 is depicted as black line, one standard deviation from mean is depicted as dashed line. (**d-e**) Surface representation of Cdc4^WD40^ indicating electrostatic potential. Blue and red indicate regions of positive and negative potential, respectively; residues showing significant cross-saturation effect are in green spheres; Sic1 peptide is in yellow. (**f**) Combined ^1^H and ^13^C chemical shifts of Cdc4^WD40^ on SCF-I2 binding. The assigned Ile and Met residues are in red, labelled with residue number; unassigned residues in pink. (**g**) Surface and ribbon representation of Cdc4^WD40^ with SCF-I2 (3MKS). SCF-I2 is in yellow, SCF-I2 binding pocket in cyan and residues showed significant chemical shift changes are in red spheres.

We determined that all allosteric pocket residues exhibited fast-to-intermediate timescale exchange dynamics (Fig. 2a,b), consistent with weak direct interaction, unlike the slow-intermediate exchange observed for some residues in the primary CPD pocket (see below). To identify potential contact residues between pSic1 and Cdc4^WD40^, we performed methyl cross-saturation experiments. Among the 13 assigned Ile and Met methyl resonances, only Ile396^P^, Ile594^A^, Ile596^A^ and Ile654 intensities were decreased significantly upon addition of pSic1 (Fig. 2c). Because cross-saturation exploits fast exchange between free and bound states^31^, we did not observe significant intensity changes for residues close to the primary CPD pocket due to slow exchange (Supplementary note 1). However, residues around a positively charged surface that includes Arg655 and Arg664 of the allosteric pocket were susceptible to cross-saturation (Fig. 2d,e). These results unambiguously identify the allosteric site as a direct second binding pocket on Cdc4^WD40^ for CPD motifs, and eliminate the alternative possibility of indirect allosteric effects transmitted from binding at the primary CPD pocket.

Arg655, Arg664 and Trp657 are the main determinants of SCF-I2 binding in the allosteric pocket^26^. Side chains of these residues form salt bridges and hydrogen bonds with the two SCF-I2 carboxylic acids and may likewise engage phosphates. The allosteric pocket appears to be contacted only by extended multisite phosphopeptides (pSic1, Sic1^20pS69/pS76/pS80^), but not short Sic1^10pT2/pT5^, Sic1^9pT45^, Sic1^9pT173^ phosphopeptides (Supplementary Fig. 3). We speculated that tethering of an extended substrate at the primary CPD pocket may increase local CPD concentration and drive interactions at the weaker-affinity allosteric site^32^. We used the program HADDOCK^33^ to dock Sic1^20pS69/pS76/pS80^ onto the allosteric pocket based on chemical shift perturbations and cross-saturation. One of the main simulated orientations placed one CPD in the allosteric pocket with potential contacts of another CPD at the primary binding pocket (Supplementary Fig. 4a,b).

IM-labelled Cdc4^WD40^ was also titrated with unlabelled SCF-I2. We observed large chemical shift perturbations close to the SCF-I2 binding pocket (Ile594^A^, Ile596^A^, Ile654 and Ile676) and also adjacent to the P−2 sub-pocket of the CPD binding interface (Met590^P^ and Ile573^P^; Fig. 2f,g). These data confirm long-range structural effects on the CPD binding pocket caused by insertion of the inhibitor at the allosteric pocket^26^. SCF-I2 also had a smaller but still significant effect on Ile385^P^ and Ile716^P^ next to the P−1 sub-pocket, not evident in the SCF-I2 bound crystal structure^26^. These results demonstrate that pSic1 and SCF-I2 have an overlapping binding surface on Cdc4^WD40^ and would therefore be expected to compete for binding at this position^34^.

### Role of the allosteric pocket in multisite recognition

**Figure 3:**
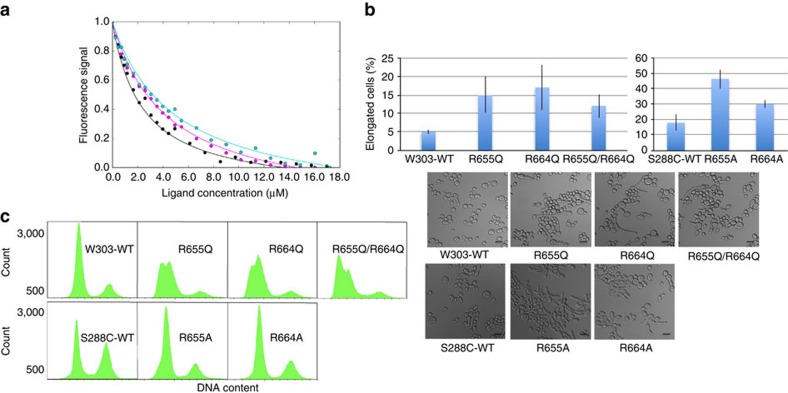
Effects of mutation of the allosteric site. (**a**) Trp fluorescence-binding curves for interactions of pSic1 and wild-type (black), Arg664Gln (cyan) and Arg655Gln Cdc4^WD40^ (magenta). (**b**) Morphology of yeast strains bearing wild-type or allosteric site mutant alleles of *CDC4* transformed with a *pGAL1-SIC1* plasmid and grown overnight on 2% galactose medium. Results are shown for the both W303 (Arg655Gln, Arg664Gln, Arg655GlnArg664Gln) and S288C (Arg655Ala and Arg664Ala) strain backgrounds. Error bars indicate s.d. for triplicate independent measurements. Scale bars, 10 μm (**c**) DNA content of wild-type and allosteric site mutant alleles of Cdc4 for the same strains shown in **b**.

We assessed the contribution of the allosteric pocket to substrate interactions by mutation of positively charged Arg655 and Arg664 residues, both of which are required for SCF-I2 inhibition^26^. We generated Arg655Gln and Arg664Gln substitution mutants to minimize disruptive steric effects on the allosteric pocket and WD40 domain. Mutation of either Arg residue resulted in four- to fivefold reduction in pSic1-Cdc4^WD40^ affinity by *in vitro* Trp fluorescence (Fig. 3a, Table 1). Importantly, these mutations did not significantly reduce CycE-Cdc4^WD40^ affinity, consistent with CycE not appreciably interacting with the allosteric pocket (Fig. 1c).

Previous mutational analysis of the allosteric pocket showed that Arg655Ala and Arg664Ala substitutions completely block SCF-I2 inhibition *in vitro*, but do not cause obvious growth or cell cycle defects *in vivo*^26^. As other nominally functional Cdc4 mutants are sensitive to Sic1 dosage and phosphorylation status^8^, we examined effects of additional Sic1 dosage on Arg655 and Arg664 mutants. While *SIC1* overexpression had little impact on mutant strain growth rates (Supplementary Fig. 4g), additional *SIC1* dosage caused accumulation of cells with elongated buds and a concomitant G1 delay (Fig. 3b,c), consistent with a mild Sic1 elimination defect. These results suggest that, while not essential, the allosteric pocket facilitates Cdc4 substrate degradation *in vivo*.

### Allosteric pocket binding weakens the primary CPD interaction

**Figure 4:**
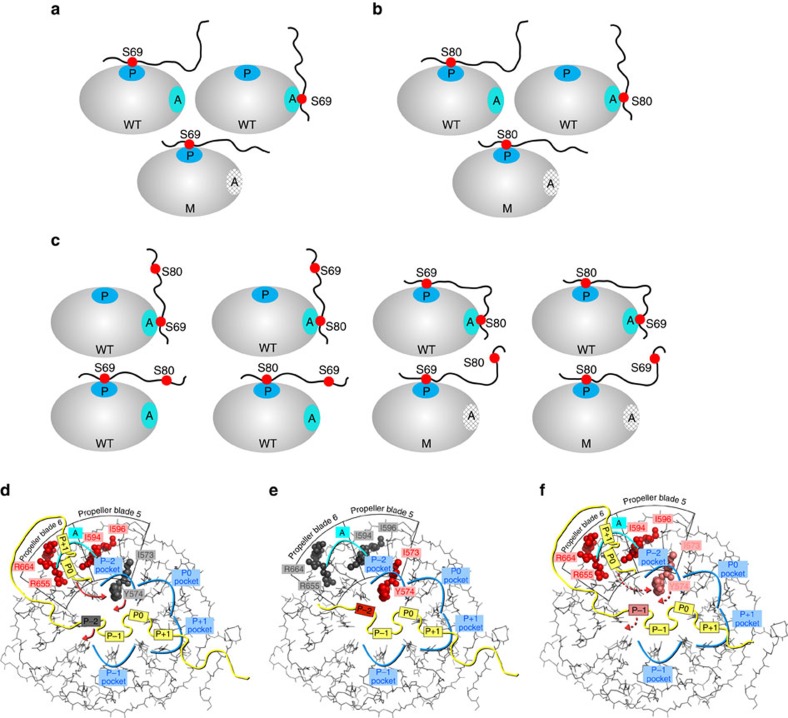
Allosteric coupling between primary and allosteric pockets for singly or doubly phosphorylated Sic1-derived peptides. (**a**–**c**) Possible binding states for (**a**) Sic1^20pS69^, (**b**) Sic1^20pS80^ and (**c**) Sic1^20pS69/pS80^ peptides to wild-type Cdc4^WD40^ and mutant (Arg664Gln) Cdc4^WD40^. (**d**-**f**) Schematic of the extent of allosteric effects caused by binding of (**d**) pSic1, (**e**) CycE peptide and (**f**) Sic1^20pS69/pS76/pS80^. For pSic1 (**d**), binding of one of the CPDs in pSic1 (P0) to the allosteric pocket (labelled A in cyan) between blade 5 and 6 induces rotation of Tyr574 (red arrows) and impairs binding in the P-2 sub-pocket (grey rectangle), demonstrated by the absence of chemical shift perturbation on Ile573 (grey spheres) on pSic1 binding. CycE peptide (**e**) does not bind the allosteric pocket, suggested by lack of chemical shift perturbation on residues around the allosteric pocket (grey spheres), and thus binding in the P-2 sub-pocket remains intact (red rectangle) and chemical shift perturbation was observed for Ile573 (red spheres). Binding of one of the CPDs in Sic1^20pS69/pS76/pS80^ (**f**) to the allosteric pocket, suggested by the chemical shift perturbation on the residues around the allosteric pocket (red spheres), has a smaller effect on the binding in the P-2 sub-pocket (red dashed arrows) compared to pSic1, demonstrating some engagement. The chemical shift perturbation for Ile573 on binding Sic1^20pS69/pS76/pS80^ peptide (light red spheres) is smaller than on binding CycE peptide (or other Sic1 peptides).

We compared affinities of Sic1 peptides phosphorylated either on Ser69 (Sic1^20pS69^) or Ser80 (Sic1^20pS80^) alone or together (Sic1^20pS69/pS80^). NMR titration of the Sic1^20pS69/pS76/pS80^ phosphopeptide perturbed chemical shifts in the primary and allosteric pockets (Supplementary Fig. 3d), suggesting that Ser69- and/or Ser80-containing CPDs can bind both pockets (Fig. 4a,b) and that the two CPDs are distant enough to enable simultaneous binding (Fig. 4c). To test this model, we computationally docked the Sic1^20pS69/pS80^ phosphopeptide onto Cdc4^WD40^ at the primary and allosteric pockets, and found that the two sites can indeed simultaneously engage with no obvious energetic strain (Supplementary Fig. 4c).

We determined phosphopeptide binding affinities for wild-type and mutant (Arg664Gln) Cdc4^WD40^ by Trp fluorescence (Supplementary Fig. 4d–f). Strikingly, the association constant for Sic1^20pS69/pS80^ binding to wild-type Cdc4^WD40^, *K*_a_(p69,p80|wt)=1.42 × 10^5^ M^−1^, was about half that for Sic1^20pS69/pS80^ binding to mutant Cdc4^WD40^, *K*_a_(p69,p80|mt)=2.67 × 10^5^ M^−1^ (see Methods, Table 2). Despite the presence of two binding pockets on wild-type Cdc4^WD40^, binding was considerably weaker than for Cdc4^WD40^ bearing only the primary CPD pocket. This result demonstrated negative allostery for a diphosphopeptide with sites that are sufficiently spaced to allow simultaneous contact with the primary and allosteric pockets. In contrast, if Cdc4^WD40^ had two independent binding sites, the expected result would be higher affinity for the wild-type due to additive contributions of both sites. We also found that the Sic1^20pS69/pS80^ *K*_a_(p69,p80|wt)=1.42 × 10^5^ M^−1^ was smaller than the sum *K*_a_(p69|wt)+*K*_a_(p80|wt)=2.07 × 10^5^ M^−1^ for singly phosphorylated peptides binding to wild-type Cdc4^WD40^. This result is also in agreement with negative allosteric coupling between the primary and allosteric pockets, although other effects that weaken combinatorial interactions at different sites may also be operative^32^. Note that the negative effect of the allosteric site on Sic1^20pS69/pS80^ diphosphopeptide binding is opposite to the decreased affinity of the allosteric site mutants for the larger pSic1^1-90^ ligand. This apparent inconsistency is resolved by accounting for long-range electrostatic contributions from the four additional CPDs in pSic1^1-90^ (see Supplementary note 4). Finally, we found that the doubly phosphorylated peptide (Sic1^20pS69/pS80^) bound mutant Cdc4^WD40^ with *K*_a_(p69,80|mt)=2.67 × 10^5^ M^−1^, approximately 1.7 times larger than the sum *K*_a_(p69|mt) +*K*_a_(p80|mt)=1.56 × 10^5^ M^−1^ for the singly phosphorylated peptides. This result is consistent with additional long-range electrostatic contributions from a second CPD in the absence of direct contact with Cdc4 (ref. 35).

### Chemical shift perturbations in the primary CPD pocket

We then examined NMR chemical shift and line-shape perturbations for signatures of negative allostery. Although population-weighted averaging and differential broadening due to exchange between states prohibits quantitative interpretation, qualitative comparisons are possible. First, we examined residues near the P−1 sub-pocket lined by Trp717 and Val384 (Supplementary Fig. 5a). CycE led to Ile385^P^ chemical shift changes dependent on peptide concentration (fast exchange), whereas two peaks were observed for Ile716^P^ (slow-intermediate exchange; Supplementary Fig. 5b). The opposite was seen for pSic1 with Ile385^P^ in slow-intermediate and Ile716^P^ in fast exchange (Supplementary Fig. 5c). These chemical shift perturbations demonstrate binding of both CycE and pSic1 in the P−1 sub-pocket, albeit with some differences.

**Figure 5:**
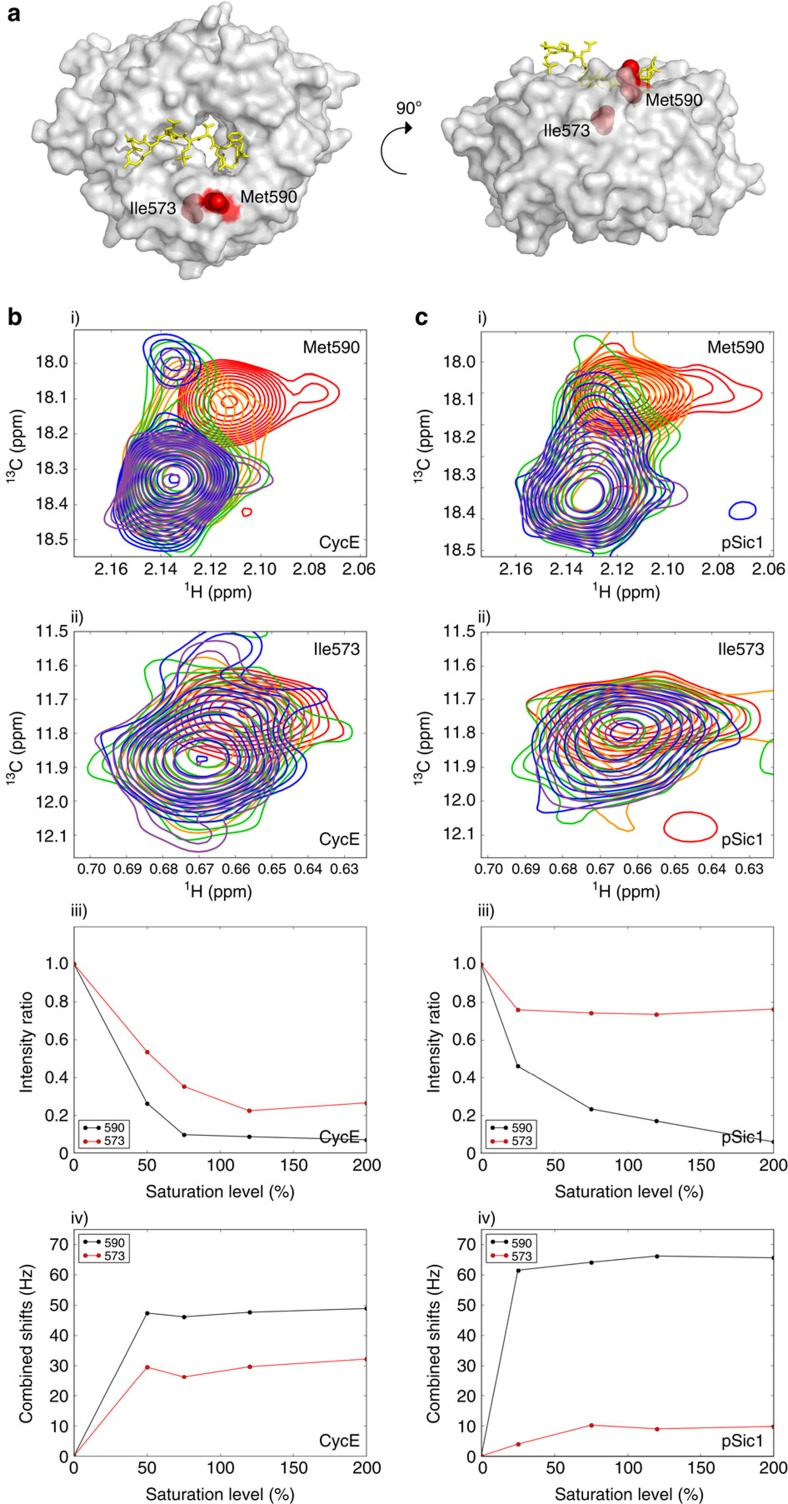
Chemical shift perturbation for residues in P-2 pocket upon CycE and pSic1 binding. (**a**) Surface representation of Cdc4^WD40^ with Sic1 peptide in yellow and residues of the P-2 pocket in red spheres. (**b**,**c**) ^1^H–^13^C TROSY HMQC spectra of residues (i) Met590 and (ii) Ile573 in the absence (red) and presence of increasing amounts (50% (orange), 75% (green), 120% (blue), 200% (purple)) of (**b**) CycE peptide or (**c**) pSic1, (iii) intensity ratios of the peaks corresponding to the unbound states and (iv) chemical shifts of the peaks corresponding to the bound states at different saturation levels expressed as a percentage of bound substrates at the given Cdc4 to substrate molar ratio.

A striking difference between pSic1 and CycE was evident for Met590^P^ and Ile573^P^ close to the P−2 sub-pocket that binds hydrophobic residues of the CPD (Fig. 5a). For both CycE and pSic1, two peaks were observed for each residue reflecting slow-intermediate exchange between free and bound states (Fig. 5b,c). However, the complexity of the NMR line-shapes suggested a dynamic equilibrium, possibly including partially engaged states. Met590^P^ is one of the most exposed side chains near the CPD pocket (Fig. 5a) and may be a general probe of substrate interactions. The loss of intensity and chemical shift perturbations for Met590^P^ in both CycE and pSic1 titrations were similar. In contrast, Ile573^P^ lies at the bottom of the P−2 sub-pocket and should be sensitive to deep engagement (Fig. 5a). While Ile573^P^ was broadened and showed chemical shift changes in the presence of CycE (Fig. 5b), neither effect was significant for pSic1 (Fig. 5c). These results suggest that the P−2 sub-pocket interacts less with pSic1 than CycE.

To investigate whether the apparent differential engagement of the P−2 pocket by pSic1 and CycE was due to the absence of preferred hydrophobic residues in P−2 positions of 8 of the 9 CPD sites of pSic1, titrations of pSic1 phosphopeptides (Sic1^10pT2/pT5^, Sic1^9pT45^, Sic1^9pT173^ and Sic1^20pS69/pS76/pS80^) were analysed. The binding of all phosphopeptides perturbed chemical shifts of Ile573^P^ (Supplementary Fig. 3), as for CycE. The Sic1^20pS69/pS76/pS80^ phosphopeptide actually had the smallest Ile573^P^ perturbation, about one third that of CycE. This was surprising since the Leu at the P−2 position of the pSer76 site is a match to the Leu/Ile consensus; if the P−2 position were the sole determinant of binding, we might expect the Sic1^20pS69/pS76/pS80^ phosphopeptide to have the largest Ile573^P^ perturbation yet this was not the case. From these results, we concluded that the lack of consensus Leu/Ile residues in P−2 positions of pSic1 CPDs cannot explain the apparent differences in pSic1 and CycE binding the P−2 pocket, and instead suggested an allosteric effect might be at play.

The allosteric inhibitor SCF-I2 is known to cause occlusion of the P−2 sub-pocket due to re orientation of a main chain Tyr residue^26^. Both pSic1 and Sic1^20pS69/pS76/pS80^ significantly perturbed residues on blade 5 (Ile594^A^, Ile596^A^ and Ile577^A^) and also caused unexpectedly small chemical shift perturbations at Ile573^P^ relative to CycE. We speculate that this NMR effect reflects decreased binding at the P−2 sub-pocket due to negative allosteric modulation of the primary CPD pocket upon simultaneous binding of another CPD site in the extended Sic1 multisite degrons (Fig. 4d–f).

### Interactions of Ash1 with Cdc4^WD40^

**Figure 6:**
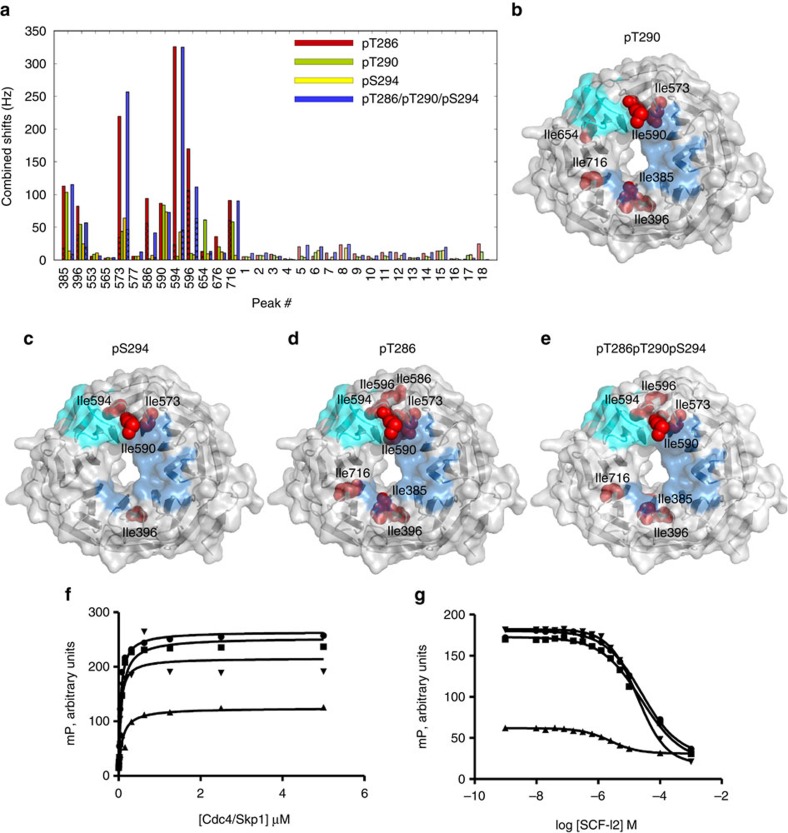
NMR titration of IM-labelled Cdc4^WD40^ with Ash1 peptides. (**a**) Combined ^1^H and ^13^C chemical shifts of Cdc4^WD40^ on binding of Ash1 peptides, with pT286 peptide in red, pT290 peptide in green, pS294 peptide in yellow, and the peptide phosphorylated on all three CPDs in blue. Assigned Ile and Met residues are labelled with residue number. Chemical shifts for the two bound states in titration with pT286 and triply phosphorylated peptides are reported on the same bars with stripes or without stripes. (**b**–**e**) Surface and ribbon representation of Cdc4^WD40^ coloured as in Fig. 1b except only residues showing significant chemical shift changes upon (**b**) pT290 peptide, (**c**) pS294 peptide, (**d**) pS286 peptide and (**e**) triply phosphorylated Ash1 peptide binding are displayed in red spheres. (**f**) Fluorescence polarization of the fluorescein-labelled Ash1 peptides (circle, pT286/pT290/pS294, square, pT286/pT290, triangle, pT290/pS294, inverted triangle doubly phosphorylated CycE peptide) in the presence of increasing concentrations of the recombinant Skp1-Cdc4 complex (mP, millipolarization unit). (**g**) Fluorescence polarization of the fluorescein-labelled Ash1 peptides (circle, pT286/pT290/pS294, square, pT286/pT290, triangle, pT290/pS294, inverted triangle, doubly phosphorylated CycE peptide) in the presence of increasing concentrations of the SCF-I2 inhibitor.

We then investigated binding of IM-labelled Cdc4^WD40^ to unlabelled phosphopeptides derived from Ash1 residues 283-299. Singly phosphorylated peptides around pThr286, pThr290 and pSer294 sites have affinities for Cdc4 of 0.9, 4.1 and 6.5 μM, respectively, and the triphosphorylated peptide has an affinity of 0.07 μM (ref. 20). Ash1^9pT290^ significantly perturbed residues close to the primary CPD pocket but not residues near the allosteric pocket (Fig. 6a,b). Ash1^9pS294^ had much less effect on the P−1 sub-pocket than Ash1^9pT290^ since Ile385^P^ and Ile716^P^ did not show significant perturbations (Fig. 6a,c). Since the P−1 and P−2 positions for the Thr290 and Ser294 sites match the CPD consensus (Supplementary Fig. 2c), the observed difference for the P−1 sub-pocket likely reflects the weaker affinity of pSer versus pThr for the P0 sub-pocket^6^. Consistently, a pSic1 phosphopeptide containing pSer at P0 (Sic1^20pS69/pS76/pS80^) also minimally perturbed the P−1 sub-pocket (Supplementary Fig. 3).

In contrast to the lower affinity Ash1 CPD motifs, Ash1^9pT286^ led to two peaks for Ile385^P^, Ile396^P^, Ile573^P^, Ile586^A^, Ile594^A^ and Ile596^A^ resonances in the fully saturated complex, diagnostic of two bound-state conformations (Supplementary Fig. 6a). In one of these, chemical shift perturbations for Ile573^P^, Ile594^A^ and Ile596^A^ were much greater than for other residues (Fig. 6a,d). Titration with the Ash1^18pT286/pT290/pS294^ triphosphopeptide resulted in similar chemical shift perturbations as for Ash1^9pThr286^ but with slightly smaller shifts for Ile396^P^ and Ile586^A^ (Fig. 6a,e, Supplementary Fig. 6b). The magnitude of shifts for residues in the allosteric pocket for these Ash1 peptides was significantly larger than for pSic1 (Fig. 1e), suggesting that the short pThr286 CPD had greater direct effects on the primary CPD pocket and also indirect effects on the allosteric pocket.

### Differential effects of SCF-I2 on Ash1 CPDs

Using fluorescence polarization^26^, we found that SCF-I2 was 10-fold more potent for inhibiting Ash1^18pT290/pS294^ binding than Ash1^18pT286/pT290^ or Ash1^18pT286/pT290/pS294^ peptides (Fig. 6f,g, Table 3). However, since the Ash1^18pT286/pT290/pS294^ bound equally well to wild-type versus Arg655Gln or Arg664Gln Cdc4^WD40^ (Supplementary Fig. 6c), we concluded the pThr286 CPD exerted an indirect allosteric effect on the SCF-I2 binding pocket. HADDOCK docking of Ash1 phosphopeptides to Cdc4^WD40^ based on chemical shift perturbations was used to provide a model for allosteric transmission. In these models, the P−2 sub-pocket of Cdc4 was occupied by the P−2 prolines of pThr290 and pSer294 CPDs but by the P−3 tryptophan of the pThr286 CPD, possibly due to predicted pi–pi interactions with Tyr594 (Supplementary Fig. 6d). Since SCF-I2 binding causes residue rearrangements near the CPD pocket, including Leu634, Val 635 and Tyr594 (Supplementary Fig. 6e), interaction between the P−3 tryptophan and Tyr594 may inhibit such rearrangements, thereby more effectively outcompeting SCF-I2 than other CPDs. These prominent indirect effects of the pThr286 Ash1 CPD on the allosteric pocket are in contrast to the minimal effects of CycE on the allosteric site and the direct allosteric site contacts made by CPDs in extended multisite ligands. The contributions of the allosteric pocket in Cdc4 may thus be modulated in a substrate-specific manner.

### Biophysical model for dynamic site exchange

**Figure 7:**
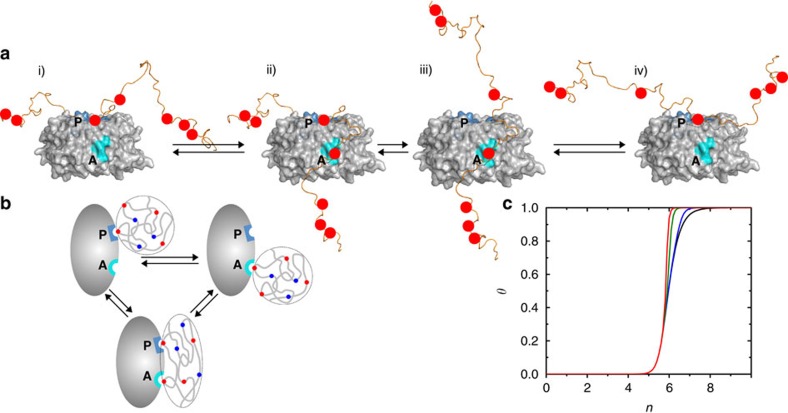
The allosteric binding pocket in Cdc4^WD40^ facilitates the dynamic exchange of CPD sites in pSic1. (**a**) Interaction of one of the CPD motifs with the high-affinity, primary (**P**) CPD-binding pocket (dark blue) (i) facilitates the binding of another CPD site to the lower affinity allosteric pocket (**A**) (cyan) (ii). Binding to the allosteric pocket weakens the engagement of pSic1 in the P-2 sub-pocket via allosteric effects (iii) and increases the probability of the rebinding of another CPD site in the primary pocket (iv). (**b**) Schematic of a possible mechanism that enhances ultrasensitivity by involving both the primary (**P**) and allosteric (**A**) binding pockets on Cdc4 (solid ellipse) in its interaction with Sic1 (chain in dashed envelope, negatively charged phosphorylated CPDs and positively charged residues are represented, respectively, by red and blue circles). The Sic1-Cdc4 bound state comprises three interconverting configurations: only **P** engaged, only **A** engaged (top drawings), and both **P** and **A** engaged (bottom drawing). The Sic1 conformations of the transiently bound configuration with both **P** and **A** engaged simultaneously are envisioned to be spatially closer to Cdc4, on average (bottom drawing), than when only **P** or only **A** is engaged (top drawings). (**c**) Fractional bound ligand *θ* versus number of phosphorylation *n* predicted by the mathematical model. The average Sic1-Cdc4 distance is taken to be 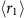
=12 Å for the **P**-only or **A**-only configuration. The ultrasensitive transition is sharper when both **P** and **A** can be engaged (colour curves) than if only **P** can be engaged (black curve). The sharpness of the transition increases with decreasing average Sic1-Cdc4 distance 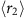
= 8 Å (blue), 6 Å (green) and 5 Å (red) for the bound configuration with both **P** and **A** engaged simultaneously (bottom drawing in **b**).

The above results suggested that dynamic exchange of the CPDs may be facilitated through negative allosteric communication between the primary and allosteric pockets. Binding of one CPD to the primary pocket would tether other CPDs in close proximity to Cdc4, and thereby enhance interactions with the low-affinity allosteric pocket (Fig. 7a, i and ii). In contrast to simple two-site binding, however, CPD binding to the allosteric pocket also weakens the CPD interaction with the primary pocket (Fig. 7a, iii), facilitating disassociation and subsequent rebinding to the primary pocket, possibly involving a previously non-engaged CPD (Fig. 7a, iv). Notably, engagement at the allosteric pocket provides a physical basis for tethering of Sic1 to Cdc4 upon transient disengagement from the primary CPD site.

In an earlier model, we rationalized the physical basis for ultrasensitivity by through-space long-range interactions between a single positively charged binding pocket on Cdc4 and the collection of negatively charged phosphates on Sic1 (ref. 35). Given evidence for CPD engagement of the allosteric pocket, we sought to ascertain whether the hypothesized polyelectrostatic basis for ultrasensitivity would hold. We extended our mathematical model to three bound configurations, under the same simplifying assumption of equivalent CPD motifs as the original model^35^ (Fig. 7b, Supplementary Fig. 7; see Supplementary note 3 for mathematical formulation). We refer to the single-point bound configurations with only the primary (**P**) pocket or only the allosteric (**A**) pocket engaged as P and A′, respectively, and the two-point bound configuration with both pockets engaged simultaneously as (P′,A). In this model, despite conformational entropy loss for two-point binding^36^, the (P′,A) configuration becomes more favourable than P or A′ when the Sic1 net charge becomes negative because the average Sic1 to Cdc4 distance for (P′,A) is smaller due to the constraint imposed on the Sic1 conformational ensemble by two-point binding, resulting in stronger electrostatic attraction between Cdc4 and non-contacting CPDs. This two-point contact model can lead to sharper ultrasensitive binding transitions than single-site models even though the transition midpoint remains essentially identical (Fig. 7c, Supplementary Fig. 7a). The critical role of non-contacting CPDs is underscored by considering an alternate model without long-range electrostatic contributions to binding, in which case ultrasensitivity is lost (Supplementary Fig. 7b). Note that the enhancement of ultrasensitivity by two-site binding does not rely on a precise degree of positive or negative allosteric coupling between the two Cdc4 sites but instead depends only on the existence of a two-site bound state to tether the Sic1 conformational ensemble closer to Cdc4 (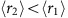). Negative coupling is, however, required for a similar transition midpoint in the one- and two-site models (Supplementary Fig. 7c). Comparing ultrasensitivity for different degrees of negative coupling between the two Cdc4 binding pockets, we found that as long as the bound Sic1 conformations are held closer on average to the Cdc4 for two-point than for one-point binding by ∼20% or more, ultrasensitivity is higher for the two-point model, even in the presence of substantial negative coupling (Supplementary Fig. 7d). Thus, the proposed ultrasensitivity enhancement effect for the two-site model is robust.

## Discussion

Multisite phosphorylation is often the basis for cellular decision-making^37^ and can control kinase priming events^38^, substrate dephosphorylation, subcellular localization and signal integration^39^. Multisite phosphorylation can lead to either linear graded responses^40^ or non-linear ultrasensitivity^6^,^11^,^12^. Sic1-Cdc4 is a well-studied example of an ultrasensitive interaction^6^,^7^,^8^,^13^,^14^. However, the molecular basis for Sic1 multisite-dependence has been elusive, in part because the consequences of substrate interactions on Cdc4 itself have never been investigated. Our assignment of 13 methyl groups positioned throughout the Cdc4 WD40 domain provides the first effective NMR probes for investigating substrate effects on Cdc4^WD40^. This approach enabled detection of unexpected direct binding interactions of Sic1 CPDs to the previously described SCF-I2 allosteric pocket, a finding with major ramifications. First, the allosteric pocket tethers Sic1 near the primary CPD pocket, curtailing Sic1 diffusion away from Cdc4 and increasing Sic1 local concentration. Second, based on affinity measurements, engagement of the allosteric pocket by CPDs weakens other CPD interactions with the primary pocket, likely by inducing rearrangement of P−2 sub-pocket residues analogous to the effect of SCF-I2. This negative allosteric effect facilitates the exchange of multiple CPD motifs, and increases the probability of rebinding another CPD site. The allosteric effect also explains the decreased chemical shift perturbations in the P−2 sub-pocket for extended multisite pSic1 substrates compared to short CycE and Sic1 phosphopeptides.

Identification of the allosteric pocket as a second direct binding determinant adds complexity to the already intricate nature of multisite Cdc4 substrate interactions. Since the sequence composition of individual CPD motifs varies considerably, some may bind to the allosteric pocket with higher affinity. Because of negative allosteric coupling between the primary CPD and allosteric pockets, CPDs that preferentially bind the allosteric pocket may have complex effects on substrate recognition. As the primary CPD and allosteric pockets are separated by ∼25 Å, simultaneous engagement can only occur if CPDs are separated by at least 7 residues. In Sic1, all but two CPD pairs meet this requirement, making many binding scenarios possible. These subtleties may affect timing of substrate degradation and confer differential susceptibility to small molecule allosteric inhibitors. We speculate that non-phosphorylated motifs in extended substrates may also engage the allosteric site to further refine substrate recognition.

Different models have been proposed for Sic1-Cdc4 ultrasensitivity. One initial proposal posited that a potential second binding pocket on Cdc4 could tether Sic1 to increase local concentration^41^. However, a simple two-site model would be dominated by overly strong thermodynamic effects, rendering high-affinity interaction dependent only on two sites, not the multiple sites observed. A kinetic model argued that, if internal CPD motion exceeded overall Sic1 diffusion, the probability of rebinding should exhibit an ultrasensitive dependence on number of CPDs^42^, but the physical basis for this effect was unknown. An entropic model postulated that multisite phosphorylation could significantly reduce conformational entropy for unbound Sic1 to favour binding^43^, but further experiments revealed no substantial decrease in Sic1 conformational disorder upon phosphorylation^14^.

Our polyelectrostatic model suggested that long-range interactions between the positively charged CPD pocket on Cdc4 and the cohort of negatively charged phosphates on Sic1 could yield an ultrasensitive transition once the overall Sic1 charge became negative^35^, analogous to other bulk electrostatic effects^44^. However, as many Sic1 lysines are dispensable for recognition^45^, charge balance alone may not explain ultrasensitivity. Regardless, it is necessary for all models that non-contacting CPDs contribute to binding such that the bound Sic1 population increases approximately exponentially with the number of phosphorylation events. Our modelling suggests that, if the allosteric pocket contributes significantly only for intermediate to large numbers of CPDs, then disabling the allosteric pocket will decrease the sharpness but not the midpoint of the ultrasensitive transition (Supplementary note 3). This predicted effect of an allosterically-coupled two-site interaction is generalizable to other mechanisms, including tethering^41^ and transient kinetic trapping^42^. To our knowledge, this form of negative allostery involving multiple biding sites within the same extended protein ligand has not been reported previously. Importantly, it has implications for the Sic1 conformational distribution that future, more comprehensive models will need to quantitatively consider (Supplementary note 4).

Allosteric communication between binding sites may be a general feature of WD40 domains, which frequently contact more than one partner on different surfaces, in some instances mediating allostery. In one example, the transducin inhibitor phosducin binds the transducin WD40 domain to induce structural rearrangements between blades 6 and 7, creating a binding pocket for the farnesyl moiety of the partner Gγ subunit^27^. Remarkably, the key allosteric pocket residues and main chain movements are virtually superimposable between transducin and Cdc4 (ref. 26). In another example, the transcriptional regulator WDR5 interacts with RbBP5 between blades 5 and 6 to allosterically regulate the methyltransferase activity of MLL1, which binds to an arginine-rich site on WDR5 (ref. 46). As shown here, binding of the same ligand to two different sites can also elicit allosteric communication and engender unusual protein recognition behaviour, vastly diversifying the output of simple binding events. Finally, we note that the Cdc4 allosteric infrastructure appears conserved in Fbw7 (ref. 26), which is a frequently mutated tumour suppressor in human cancer^22^. Since many Fbw7 substrates are multisite phosphorylated^22^, it will be of considerable interest to determine whether allosteric coupling influences Fbw7 substrate recognition, and whether substrate-selective Fbw7 allosteric inhibitors can be developed.

## Methods

### Protein production and purification

Cdc4^WD40^ (residues 367-744) was expressed from pET-Sumo (Invitrogen), isolated by Ni-nitrilotriacetate (NTA) affinity chromatography, digested with Ulp protease, and further purified by Ni-NTA and Superdex 200 chromatography^8^. Mutations to Cdc4^WD40^ in pETSumo vector were introduced using the Quickchange (Stratagene) approach and verified by DNA sequencing. For the NMR experiments, *Escherichia coli* BL21(DE3) codonPlus cells were transformed with pETSumo plasmid encoding Cdc4^WD40^ and were grown in 99% D2O minimal medium. To achieve methyl labelling of the Ile-δ1-[^13^CH_3_] and Met-ɛ-[^13^CH_3_] (referred to as IM-labelled Cdc4^WD40^ that is U-[^15^N, ^2^H], Ileδ1-[^13^CH_3_], Metɛ-[^13^CH_3_]) the media was supplemented with 50 mg l^−1^ alpha-ketobutyric acid ([methyl-^13^CHD_2_])^47^ and 100 mg l^−1^ L-methionine (methyl-^13^CH_3_)^48^,^49^ 1 h before protein induction and was purified as described above^8^. Sic1 was expressed as a GST fusion protein in *Escherichia coli* BL21(DE3) codon plus cells. The GST-Sic1 protein was purified by using glutathione–Sepharose affinity chromatography. After digestion with TEV protease, GST was removed by using anion exchange chromatography. Sic1 was further purified to homogeneity by using a Superdex 75 column (Amersham Biosciences). Sic1 was then phosphorylated with immobilized Cln2-Cdc28 kinase from baculovirus-infected Sf9 cells^13^.

### NMR spectroscopy

*Assignment of Ile and Met residues in Cdc4^WD40^*. All NMR experiments were performed at 10 °C on Varian Inova 500 and 800 MHz spectrometers. Methyl-TROSY spectra were recorded on per-deuterated protein samples dissolved in 100% D_2_O, 50 mM Na_2_HPO_4_, pH 8.0, 150 mM NaCl, 2 mM DTT. In order to assign the methyl residues, a total of 15 Ile to Val or Met to Val point mutations were made (I385V, I573V, M590V, I716V, I676V, I594V, I396V, I404V, I553V, I565V, I586V, I596V, I654V and I577V), which resulted in unambiguous assignment of 1 Met and 13 Ile residues. (I404V mutation led to a spectrum of very poor quality that could not be used for assignment.) All spectra were processed using the program NMRPipe^50^ and the spectra were analysed using SPARKY (T.D. Goddard and D.G. Kneller, SPARKY 3 (University of California, San Francisco).

*NMR chemical shift perturbations*. To monitor the interaction of Cdc4^WD40^ with different partners, 2D ^13^C–^1^H TROSY HMQC spectra were recorded at 10 °C on 200 μM IM-Cdc4^WD40^ sample for increasing concentrations of pSic1, CycE and Ash1 peptides and SCF-I2 inhibitor. Chemical shift changes that were greater than one standard deviation from the mean were considered significant. Chemical shift perturbations (CSPs) were calculated from the relations


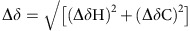


where Δ*δ* is the chemical shift change between the apo and the fully saturated states of the protein.

*Methyl-utilizing cross-saturation experiment*. The cross-saturation (CS) experiment was carried out as described in Takahashi *et al*.^31^ on a 300 μM IM-labelled Cdc4^WD40^ sample with 600 μM pSic in 100% D_2_O, 50 mM Na_2_HPO_4_, pH 8.0, 150 mM NaCl, 2 mM DTT buffer at 10 °C. Saturation of the aromatic protons in Sic1 was done by a WURST-2 adiabatic pulse with a maximum radiofrequency (RF) amplitude of 0.19 kHz. The saturation frequency was set to 7.0 with a saturation time of 1.5 s. As the saturation scheme was highly selective for the aromatic proton region, the irradiation did not affect the intensity of the pSic1 amide region. However, to ensure that CS effects seen were not transmitted by spin diffusion, a control experiment was performed in which the saturation frequency was set to −5 p.p.m. CS effects are presented as ratios of (*I*_irr_/*I*_0_) with Cdc4 and (*I*_irr_/*I*_0_) without Cdc4 [(*I*_irr_/*I*_0_)_Cdc4_/(*I*_irr_/*I*_0_)_woCdc4_].

### Molecular docking

Models for Cdc4^WD40^-Ash1 and Cdc4^WD40^-Sic1^20pS69/pS76/pS80^ complexes were obtained using the molecular docking program HADDOCK 2.1 (ref. 51), based on chemical shift perturbations for Cdc4^WD40^. Active and passive residues were selected based on the strategy outlined in ref. 33 and see also Supplementary Note 2. Docked structures, corresponding to the 200 best solutions with the lowest intermolecular energies, were generated and clustered based on a backbone RMSD tolerance of 1.0 Å. The quality of the structural ensemble was assessed using the PROCHECK program^52^.

### Isothermal titration calorimetry (ITC)

ITC experiments were performed using VP-ITC and iTC200 microcalorimeters (MicroCal Inc., Northampton, MA) at 20 °C with the sample cell containing 30 μM Cdc4^WD40^ and the syringe loaded with 100 μM pSic1 or 200 μM SCF-I2. The samples were dialysed in buffer containing 50 mM Na_2_HPO_4_, pH 7.2, 150 mM NaCl, 2 mM β-mercaptoethanol. Runs were performed using 13–20 injections at an interval of 120 s and each binding experiment was repeated twice. Using the Origin software provided by MicroCal Inc., heat associated with each injection was integrated and thermodynamic parameters were extracted by fitting the data to a single-site binding model.

### Yeast strains and cell culture

Wild type or mutant *CDC4* (Arg655Gln, Arg664Gln, Arg655Gln/Arg664Gln) alleles under control of the native CDC4 promoter were introduced on a <*CDC4 TRP CEN*> plasmid into a *cdc4ΔHIS3* strain in either W303 (*MATa ade2-1 can1-100 his3-11,15 leu2-3, 112 trp1-1 ura3 GAL11 psi+*) or S288C (BY4741 *MATa his3Δ1 leu2Δ0 met15Δ0 ura3Δ0*) backgrounds. Each strain was also co-transformed with a <*GAL1-SIC1 LEU CEN*> plasmid to allow conditional high level expression of *SIC1*. For microscopy and FACS analysis of DNA content, cells were grown to early log phase and induced overnight in selective galactose medium before imaging and staining with Sytox green dye^8^. For spot dilution assays, strains were cultured for one day in SC-Trp-Leu 2% raffinose liquid medium, then spotted in 10-fold dilutions onto SC-Trp-Leu 2% glucose or SC-Trp-Leu 2% raffinose 2% galactose plates and grown at 30 °C for three days.

### Intrinsic Trp fluorescence measurements

The binding affinities of three different peptides (Sic1^20pS69^, Sic1^20pS80^ and Sic1^20pS69/S80^) for wild-type and mutant (Arg664Gln) Cdc4^WD40^ were measured by intrinsic Trp fluorescence-binding experiments. The measurements were carried out by using an ATF 105 spectrometer (AVIV Instruments, Lakewood, NJ) equipped with an automatic titrator. The binding of the peptide fragments were monitored by using intrinsic tryptophan fluorescence with an excitation wavelength of 298 nm and an emission wavelength of 366 nm. All measurements were performed in 50 mM Tris, 150 mM NaCl, 5 mM DTT, pH 7.5 at 20 °C. The concentration of Cdc4 was 0.5 μM for all measurements^35^. Dissociation constants were determined by assuming 1:1 protein stoichiometry, given that the allosteric site interaction is not detectably perturbed by singly phosphorylated CPD peptides. Association constants were calculated using the formula *K*_a_=1/*K*_d_. Prism 4.0 software (GraphPad Software, San Diego, CA) was used for statistical analysis (non-paired Student t test), and *P* values <0.05 were considered significant. Each experiment was repeated at least three times.

### Fluorescence polarization assay

Fluorescence polarization assays containing 0.21 μM Skp1-Cdc4 complex and 10 nM fluorescently labelled cyclin E-derived or Ash1-derived phosphopeptides in 10 mM HEPES (pH 7.5), 40 mM NaCl, 0.1 mg ml^−1^ BSA and 1 mM DTT in a final volume of 23.5 μl per well were performed on an Analyst HT plate reader (Molecular Devices Corp., Sunnyvale CA). Samples were excited at 485 nm and emission was read at 535 nm (ref. 26).

### Mathematical model

We developed a variation of the mean-field electrostatic model^35^. Whereas the original model assumed only one Sic1 binding pocket on Cdc4, the model here embodies both a primary (**P**) and an allosteric (**A**) binding pocket. Accordingly, the Sic1-Cdc4 bound state consisted of three different bound configurations. To provide rationalization for the present experimental data, contact energies associated with the two binding pockets were chosen to represent experimentally observed trends. Additional energetic parameters that were likely unrealistic were also considered to explore alternate hypothetical binding scenarios and to assess the robustness of the proposed mechanism. Details are provided in the Supplementary Note 3.

### Data availability

The data that support the findings of this study are available from the corresponding author upon request.

## Additional information

**How to cite this article:** Csizmok, V. *et al*. An allosteric conduit facilitates dynamic multisite substrate recognition by the SCF^Cdc4^ ubiquitin ligase. *Nat. Commun.* **8,** 13943 doi: 10.1038/ncomms13943 (2017).

**Publisher's note:** Springer Nature remains neutral with regard to jurisdictional claims in published maps and institutional affiliations.

## Supplementary Material

Supplementary Information Supplementary Figures, Supplementary Notes and Supplementary References

## Figures and Tables

**Table 1:** Binding affinities of pSic1 and CycE for wild-type and allosteric site mutants of Cdc4^WD40^.

***K***_**d**_ **(μM)**	**pSic1 (1-90)**	**CycE**
wt Cdc4^WD40^	1.30±0.28	2.21±0.6
R655Q Cdc4^WD40^	4.16±0.11	2.90±0.48
R664Q Cdc4^WD40^	4.96±0.337	1.47±0.09

Results are the average of at least three individual fluorescence-binding experiments with s.d. of all measurements reported.

**Table 2:** Binding affinities and calculated association constants of singly and doubly phosphorylated Sic1 peptides for wild-type and allosteric site mutant Cdc4^WD40^.

	**Sic1**^**20pS69**^	**Sic1**^**20pS80**^	**Sic1**^**20pS69/S80**^
*K*_d_ (μM)			
wt Cdc4^WD40^	10.3±0.32	9.13±0.54	7.06±0.70
R664Q Cdc4^WD40^	19.5±0.08	9.58±1.08	3.74±0.59
*K*_a_ (M^−1^)			
wt Cdc4^WD40^	9.76±0.31E+04	1.10±0.06E+05	1.42±0.14E+05
R664Q Cdc4^WD40^	5.14±0.03E+04	1.04±0.12E+05	2.67±0.42E+05

Results are the average of at least three individual fluorescence-binding experiments with s.d. of all measurements reported.

**Table 3:** Calculated dissociation constant (*K*_d_) and inhibition constant (*K*_i_) values from fluorescence polarization measurements for Ash1-derived peptides.

**Ash1 peptides**	**Phosphorylated residues**	***K***_**d**_ **(μM)**	***K***_**i**_ **(μM)**
AWSIpTPPVpTPPMpSPPTNR	T286, T290, S294	0.046	25.0
AWSIpTPPVpTPPMSPPTNR	T286, T290	0.069	25.0
AWSITPPVpTPPMpSPPTNR	T290, S294	0.082	2.4

## References

[b1] Stirnimann C. U., Petsalaki E., Russell R. B. & Muller C. W. WD40 proteins propel cellular networks. Trends Biochem. Sci. 35, 565–574 (2010). 20451393 10.1016/j.tibs.2010.04.003

[b2] Willems A. R., Schwab M. & Tyers M. A hitchhiker's guide to the cullin ubiquitin ligases: SCF and its kin. Biochim. Biophys. Acta 1695, 133–170 (2004). 15571813 10.1016/j.bbamcr.2004.09.027

[b3] Petroski M. D. & Deshaies R. J. Function and regulation of cullin-RING ubiquitin ligases. Nat. Rev. Mol. Cell Biol. 6, 9–20 (2005). 15688063 10.1038/nrm1547

[b4] Skaar J. R., Pagan J. K. & Pagano M. Mechanisms and function of substrate recruitment by F-box proteins. Nat. Rev. Mol. Cell Biol. 14, 369–381 (2013). 23657496 10.1038/nrm3582 PMC3827686

[b5] Verma R. . Phosphorylation of Sic1p by G1 Cdk required for its degradation and entry into S phase. Science 278, 455–460 (1997). 9334303 10.1126/science.278.5337.455

[b6] Nash P. . Multisite phosphorylation of a CDK inhibitor sets a threshold for the onset of DNA replication. Nature 414, 514–521 (2001). 11734846 10.1038/35107009

[b7] Orlicky S., Tang X., Willems A., Tyers M. & Sicheri F. Structural basis for phosphodependent substrate selection and orientation by the SCF^Cdc4^ ubiquitin ligase. Cell 112, 243–256 (2003). 12553912 10.1016/s0092-8674(03)00034-5

[b8] Tang X. . Composite low affinity interactions dictate recognition of the cyclin-dependent kinase inhibitor Sic1 by the SCF^Cdc4^ ubiquitin ligase. Proc. Natl Acad. Sci. USA 109, 3287–3292 (2012). 22328159 10.1073/pnas.1116455109 PMC3295303

[b9] Serber Z. & Ferrell J. E. Jr Tuning bulk electrostatics to regulate protein function. Cell 128, 441–444 (2007). 17289565 10.1016/j.cell.2007.01.018

[b10] Ha S. H. & Ferrell J. E. Jr Thresholds and ultrasensitivity from negative cooperativity. Science 352, 990–993 (2016). 27174675 10.1126/science.aad5937 PMC5184821

[b11] Ferrell J. E. Jr Tripping the switch fantastic: how a protein kinase cascade can convert graded inputs into switch-like outputs. Trends Biochem. Sci. 21, 460–466 (1996). 9009826 10.1016/s0968-0004(96)20026-x

[b12] Salazar C. & Hofer T. Allosteric regulation of the transcription factor NFAT1 by multiple phosphorylation sites: a mathematical analysis. J. Mol. Biol. 327, 31–45 (2003). 12614606 10.1016/s0022-2836(03)00085-8

[b13] Mittag T. . Dynamic equilibrium engagement of a polyvalent ligand with a single-site receptor. Proc. Natl Acad. Sci. USA 105, 17772–17777 (2008). 19008353 10.1073/pnas.0809222105 PMC2582940

[b14] Mittag T. . Structure/function implications in a dynamic complex of the intrinsically disordered Sic1 with the Cdc4 subunit of an SCF ubiquitin ligase. Structure 18, 494–506 (2010). 20399186 10.1016/j.str.2010.01.020 PMC2924144

[b15] Hao B., Oehlmann S., Sowa M. E., Harper J. W. & Pavletich N. P. Structure of a Fbw7-Skp1-cyclin E complex: multisite-phosphorylated substrate recognition by SCF ubiquitin ligases. Mol. Cell 26, 131–143 (2007). 17434132 10.1016/j.molcel.2007.02.022

[b16] Koivomagi M. . Cascades of multisite phosphorylation control Sic1 destruction at the onset of S phase. Nature 480, 128–131 (2011). 21993622 10.1038/nature10560 PMC3228899

[b17] Welcker M. & Clurman B. E. Fbw7/hCDC4 dimerization regulates its substrate interactions. Cell Div. 2, 7 (2007). 17298674 10.1186/1747-1028-2-7 PMC1802738

[b18] Welcker M. . Fbw7 dimerization determines the specificity and robustness of substrate degradation. Genes Dev. 27, 2531–2536 (2013). 24298052 10.1101/gad.229195.113 PMC3861666

[b19] Tang X. . Suprafacial orientation of the SCF^Cdc4^ dimer accommodates multiple geometries for substrate ubiquitination. Cell 129, 1165–1176 (2007). 17574027 10.1016/j.cell.2007.04.042

[b20] Liu Q. . SCF^Cdc4^ enables mating type switching in yeast by cyclin-dependent kinase-mediated elimination of the Ash1 transcriptional repressor. Mol. Cell Biol. 31, 584–598 (2011). 21098119 10.1128/MCB.00845-10 PMC3028614

[b21] Duda D. M. . Structural regulation of cullin-RING ubiquitin ligase complexes. Curr. Opin. Struct. Biol. 21, 257–264 (2011). 21288713 10.1016/j.sbi.2011.01.003 PMC3151539

[b22] Davis R. J., Welcker M. & Clurman B. E. Tumor suppression by the Fbw7 ubiquitin ligase: mechanisms and opportunities. Cancer Cell 26, 455–464 (2014). 25314076 10.1016/j.ccell.2014.09.013 PMC4227608

[b23] Smock R. G. & Gierasch L. M. Sending signals dynamically. Science 324, 198–203 (2009). 19359576 10.1126/science.1169377 PMC2921701

[b24] Gunasekaran K., Ma B. & Nussinov R. Is allostery an intrinsic property of all dynamic proteins? Proteins 57, 433–443 (2004). 15382234 10.1002/prot.20232

[b25] Hilser V. J. Biochemistry. An ensemble view of allostery. Science 327, 653–654 (2010). 20133562 10.1126/science.1186121 PMC5822688

[b26] Orlicky S. . An allosteric inhibitor of substrate recognition by the SCF(Cdc4) ubiquitin ligase. Nat. Biotechnol. 28, 733–737 (2010). 20581844 10.1038/nbt.1646 PMC4445864

[b27] Loew A., Ho Y. K., Blundell T. & Bax B. Phosducin induces a structural change in transducin beta gamma. Structure 6, 1007–1019 (1998). 9739091 10.1016/s0969-2126(98)00102-6

[b28] Verma R., Feldman R. M. & Deshaies R. J. SIC1 is ubiquitinated *in vitro* by a pathway that requires CDC4, CDC34, and cyclin/CDK activities. Mol. Biol. Cell 8, 1427–1437 (1997). 9285816 10.1091/mbc.8.8.1427 PMC276167

[b29] Rosenzweig R. & Kay L. E. Bringing dynamic molecular machines into focus by methyl-TROSY NMR. Annu. Rev. Biochem. 83, 291–315 (2014). 24905784 10.1146/annurev-biochem-060713-035829

[b30] Tugarinov V., Hwang P. M., Ollerenshaw J. E. & Kay L. E. Cross-correlated relaxation enhanced ^1^H-^13^C NMR spectroscopy of methyl groups in very high molecular weight proteins and protein complexes. J. Am. Chem. Soc. 125, 10420–10428 (2003). 12926967 10.1021/ja030153x

[b31] Takahashi H. . Utilization of methyl proton resonances in cross-saturation measurement for determining the interfaces of large protein-protein complexes. J. Biomol. NMR 34, 167–177 (2006). 16604425 10.1007/s10858-006-0008-8

[b32] Zhou H. X. Quantitative account of the enhanced affinity of two linked scFvs specific for different epitopes on the same antigen. J. Mol. Biol. 329, 1–8 (2003). 12742013 10.1016/s0022-2836(03)00372-3

[b33] Dominguez C., Boelens R. & Bonvin A. M. HADDOCK: a protein-protein docking approach based on biochemical or biophysical information. J. Am. Chem. Soc. 125, 1731–1737 (2003). 12580598 10.1021/ja026939x

[b34] Vauquelin G. & Charlton S. J. Exploring avidity: understanding the potential gains in functional affinity and target residence time of bivalent and heterobivalent ligands. Br. J. Pharmacol. 168, 1771–1785 (2013). 23330947 10.1111/bph.12106 PMC3623049

[b35] Borg M. . Polyelectrostatic interactions of disordered ligands suggest a physical basis for ultrasensitivity. Proc. Natl Acad. Sci. USA 104, 9650–9655 (2007). 17522259 10.1073/pnas.0702580104 PMC1887549

[b36] Song J., Ng S. C., Tompa P., Lee K. A. W. & Chan H. S. Polycation-pi interactions are a driving force for molecular recognition by an intrinsically disordered oncoprotein family. PLoS Comput. Biol. 9, e1003239 (2013). 24086122 10.1371/journal.pcbi.1003239 PMC3784488

[b37] Cohen P. The regulation of protein function by multisite phosphorylation--a 25 year update. Trends Biochem. Sci. 25, 596–601 (2000). 11116185 10.1016/s0968-0004(00)01712-6

[b38] McGrath D. A. . Cks confers specificity to phosphorylation-dependent CDK signaling pathways. Nat. Struct. Mol. Biol. 20, 1407–1414 (2013). 24186063 10.1038/nsmb.2707 PMC4242096

[b39] Schweiger R. & Linial M. Cooperativity within proximal phosphorylation sites is revealed from large-scale proteomics data. Biol. Direct. 5, 6 (2010). 20100358 10.1186/1745-6150-5-6 PMC2828979

[b40] Holmberg C. I., Tran S. E., Eriksson J. E. & Sistonen L. Multisite phosphorylation provides sophisticated regulation of transcription factors. Trends Biochem. Sci. 27, 619–627 (2002). 12468231 10.1016/s0968-0004(02)02207-7

[b41] Deshaies R. J. & Ferrell J. E. Jr Multisite phosphorylation and the countdown to S phase. Cell 107, 819–822 (2001). 11779457 10.1016/s0092-8674(01)00620-1

[b42] Klein P., Pawson T. & Tyers M. Mathematical modeling suggests cooperative interactions between a disordered polyvalent ligand and a single receptor site. Curr. Biol. 13, 1669–1678 (2003). 14521832 10.1016/j.cub.2003.09.027

[b43] Lenz P. & Swain P. S. An entropic mechanism to generate highly cooperative and specific binding from protein phosphorylations. Curr. Biol. 16, 2150–2155 (2006). 17084700 10.1016/j.cub.2006.09.013

[b44] Strickfaden S. C. . A mechanism for cell-cycle regulation of MAP kinase signaling in a yeast differentiation pathway. Cell 128, 519–531 (2007). 17289571 10.1016/j.cell.2006.12.032 PMC1847584

[b45] Petroski M. D. & Deshaies R. J. Context of multiubiquitin chain attachment influences the rate of Sic1 degradation. Mol. Cell 11, 1435–1444 (2003). 12820958 10.1016/s1097-2765(03)00221-1

[b46] Avdic V. . Structural and biochemical insights into MLL1 core complex assembly. Structure 19, 101–108 (2011). 21220120 10.1016/j.str.2010.09.022

[b47] Goto N. K., Gardner K. H., Mueller G. A., Willis R. C. & Kay L. E. A robust and cost-effective method for the production of Val, Leu, Ile (δ 1) methyl-protonated 15N-, 13C-, 2H-labeled proteins. J. Biomol. NMR 13, 369–374 (1999). 10383198 10.1023/a:1008393201236

[b48] Gelis I. . Structural basis for signal-sequence recognition by the translocase motor SecA as determined by NMR. Cell 131, 756–769 (2007). 18022369 10.1016/j.cell.2007.09.039 PMC2170882

[b49] Religa T. L., Sprangers R. & Kay L. E. Dynamic regulation of archaeal proteasome gate opening as studied by TROSY NMR. Science 328, 98–102 (2010). 20360109 10.1126/science.1184991

[b50] Delaglio F. . NMRPipe: a multidimensional spectral processing system based on UNIX pipes. J. Biomol. NMR. 6, 277–293 (1995). 8520220 10.1007/BF00197809

[b51] de Vries S. J., van Dijk M. & Bonvin A. M. The HADDOCK web server for data-driven biomolecular docking. Nat. Protoc. 5, 883–897 (2010). 20431534 10.1038/nprot.2010.32

[b52] Laskowski R. A., MacArthur M. W., Moss D. S. & Thornton J. M. PROCHECK: A program to check the stereochemical quality of protein structures. J. Appl. Cryst. 26, 283–291 (1993).

