# Supplementary material for: An allosteric conduit facilitates dynamic multisite substrate recognition by the SCFCdc4 ubiquitin ligase
Source: Nat Commun. 2017 Jan 3;8:13943. doi: 10.1038/ncomms13943 (PMC5216119; doi:10.1038/ncomms13943)
Supplement: Supplementary Information — Supplementary Figures, Supplementary Notes and Supplementary References [file ncomms13943-s1.pdf]

Supplementary Information

Supplementary Note 1

<u>NMR spectroscopy</u>

Under conditions of fast exchange on the NMR timescale, when the rate of exchange is faster than the frequency difference between the two states, a single peak is observed at a population-weighted averaged resonance frequency and titration of a binding partner leads to chemical shift perturbations reflective of the relative amounts of free and bound states. These are dependent on the amount of added protein/peptide partner until saturation, with the last titration point being fully saturated; the data for this saturation point are reported in this study. Under conditions of slow exchange on the NMR timescale, when the rate of exchange is slower than the frequency difference, two peaks are observed with their integrated intensities corresponding to the populations of free and bound states and straightforward chemical shift differences between the two states, which are independent of concentration. For intermediate exchange, either the single peak becomes broad as ligand is added and migrates from the free to the bound state (fast-intermediate) or two broadened peaks are observed (slow-intermediate). The difference in chemical shift or frequency between the free and bound states depends on the change in electronic environment caused by structural changes upon binding or direct atomic contacts as well as the degree of engagement of residues in the substrate with the binding interface, noting that a protein can be macroscopically bound without all atoms being similarly immobilized on the surface. More complex dynamic exchange involving partially engaged bound states are thus possible, potentially resulting in more broadening. Interactions with the $Cdc4^{WD40}$ led to slow-intermediate or fast exchange behavior due to a range of impacts on the electronic environment and likely complex multi-state equilibria. Resonance intensities in general decrease upon binding of the ~10 kDa pSic1 and smaller peptide fragments to the ~40 kDa $Cdc4^{WD40}$ with additional losses of intensities due to the intermediate exchange behavior resulting from these multiple bound states.

Methyl-utilizing cross-saturation experiment

This method exploits fast exchange between free and bound states of the complex. In our experimental setup, $Cdc4^{WD40}$ was completely saturated with pSic1, therefore we did not observe significant intensity changes for residues in the primary pocket due to their slow exchange. In our earlier publication[1], a different experiment, namely transfer cross-saturation (TCS), was used to map the interaction between the protonated Cdc4-Skp1 and deuterated pSic1, and significant TCS effects

were detected for multiple phosphorylation sites. The pSic1:Cdc4 molar ratio in these previous experiments was 26:1; therefore the pSic1 was less than 5 % saturated by Cdc4.

Supplementary Note 2

Molecular docking

For the Cdc4^{WD40}-Ash1 and Cdc4-Sic1$^{20pS69/pS80}$ complexes we used the previously published Cdc4 structure in complex with CycE peptide (PDB entry 1NEX, chain D[2]); structures of the three Ash1 peptides and Sic1$^{20pS69/pS80}$ were generated by Crystallography & NMR system 1.2 (CNS). Active residues for Cdc4 were those which mediate interaction with CycE and Sic1$^{20pS69/pS76/pS80}$ peptide in the complexes solved by X-ray crystallography, namely Leu634, Met590, Tyr574, Arg572, Tyr548, Arg534, Arg467, Arg485, Thr465, thr441, Trp426, Val384, Trp717. Active residues for the Ash1 and Sic1$^{20pS69/pS80}$ peptides were the residues in the P-2, P-1, P0, P+1 and P+2 positions. Residues adjacent to active residues were chosen as passive residues. For the Cdc4 ^{WD40}-Sic1$^{20pS69/pS76/pS80}$ complex we used the previously published Cdc4 structure in complex with SCF-I2 (PDB entry 3MKS, chain D[3]) because in this structure the secondary binding pocket is induced by separation of blades 5 and 6. The structure of Sic1$^{20pS69/pS76/pS80}$ was generated by CNS. Active residues for Cdc4 in this case were selected based on NMR titration data, the Trp fluorescence binding measurement with Cdc4 mutants, and based on the X-ray structure of Cdc4 in complex with SCF-I2. The selected residues were Arg655, Trp657, Arg664, Ile594 and Ile596. Active residues for the Sic1$^{20pS69/pS76/pS80}$ peptide were the residues in the P-2, P-1, P0, P+1 and P+2 positions. Residues adjacent to active residues were chosen as passive residues. All ambiguous interaction restraints, AIR[4], were defined with a maximum effective distance of 2 Å. The docking was initiated with random starting orientations of Cdc4 and Ash1 or Sic1$^{20pS69/pS76/pS80}$ peptides separated by a minimum of 25 Å, with each molecule rotated randomly around its center of mass. Next, it was followed by four cycles of rotational minimization in which each molecule is allowed to rotate in turn and two cycles of rotational and translational rigid body minimization in which each molecule and associated solvent is treated as one rigid body. The best 200 structures after rigid body docking were subjected to a semi-flexible simulated annealing (SA) in torsion angle space whereby sidechains and loops were allowed to move. In the final step, the structures obtained after the semi-flexible simulated annealing with refined in an explicit solvent layer.

Supplementary Note 3

Details of mathematical model

As outlined in the main text, the present model is an extension of the model of Borg et al[5]. The less-than-additive binding data for a peptide phosphorylated on two sites compared to the constituent singly phosphorylated peptides, i.e., Ka(p69,p80|wt) < Ka(p69|wt) + Ka(p80|wt), provided evidence for negative allostery between the two binding pockets in wild-type Cdc4 in that overall binding is weakened by the *possibility* of simultaneous binding to the two pockets. Although only overall binding was measured – the affinity for simultaneous binding was not determined separately and thus theory[6] that requires the latter data as input cannot be applied –, this experimental finding suggested that our previous mathematical model[5] may be refined by incorporating of non-additive contact energies. The resulting model described below showed that Sic1-Cdc4 ultrasensitivity can be enhanced by binding to both the primary and allosteric pockets despite a certain degree of negative allostery provided, as it is physically likely, that additional binding to the allosteric pocket would, on average, hold the Sic1 conformational ensemble in closer proximity to Cdc4 (Fig. 7c, Supplementary Fig. 7a, d).

Here we follow the rationale, basic setup, and notation used previously by Borg et al[5]. The original model allows only for a single Cdc4 binding pocket for Sic1. Accordingly, the partition function for the bound Sic1-Cdc4 complex was given by

$$Q_c^{(b)} \propto n \exp\left[-\frac{E_b + q_r q_l e^{-\alpha\langle r\rangle}/(\varepsilon_d\langle r\rangle)}{k_B T}\right] = \exp\left(-\frac{E_b}{k_B T}\right)\exp\left[-\frac{q_r q_l e^{-\alpha\langle r\rangle}/(\varepsilon_d\langle r\rangle)}{k_B T}\right],$$

[S1]

where n is the number of phosphorylations, E_b is the contact energy between the directly engaged phosphorylated CPD on the Sic1 ligand and the binding pocket of the Cdc4 receptor, q_r and q_l are the electric charges, respectively, on the Cdc4 receptor and Sic1 ligand, α is a Debye-Hückel screening parameter, $\langle r\rangle$ is an effective Sic1-Cdc4 separation for electrostatic interactions, ε_d is dielectric constant, k_B is Boltzmann constant and T is absolute temperature. When the bound state is generalized to encompass three different bound configurations (Fig. 7b), the partition function for the complex becomes

$$Q_c^{(b)} \propto n\left[\exp\left(-\frac{E_b^P}{k_B T}\right) + \exp\left(-\frac{E_b^{A'}}{k_B T}\right)\right]\exp\left[-\frac{q_r q_l e^{-\alpha\langle r_1\rangle}}{\varepsilon_d\langle r_1\rangle k_B T}\right] + n(n-1)\exp\left(-\frac{E_b^{P'}+E_b^{A}}{k_B T}\right)\exp\left[-\frac{q_r q_l e^{-\alpha\langle r_2\rangle}}{\varepsilon_d\langle r_2\rangle k_B T}\right]\langle e^{\Delta S_2}\rangle .$$

[S2]

Here E_b^P and $E_b^{A'}$ are, respectively, the contact energy between a directly engaged phosphorylated CPD

with the primary pocket and the allosteric pocket when only one of the pockets is engaged (P or A′), whereas $E_{\mathrm{b}}^{\mathrm{P}'}$ and $E_{\mathrm{b}}^{\mathrm{A}}$ are, respectively, the contact energy between a directly engaged phosphorylated CPD with the primary pocket and another directly engaged phosphorylated CPD with the allosteric pocket when both pockets are engaged simultaneously (P′,A). The distances $\langle r_1 \rangle$ and $\langle r_2 \rangle$ are, respectively, the effective Sic1-Cdc4 electrostatic distance in the single-point and two-point bound configurations. ΔS_2 is the change in bound Sic1 conformational entropy caused by fixing two points along the chain for (P′,A) compared with fixing one point for P or A′. This conformational entropy change depends in general on the separation between the two directly engaged CPDs. For simplicity, however, results shown in the present work are obtained using an average factor $\langle e^{\Delta S_2} \rangle$ that accounts approximately for the reduction in the number of conformations caused by two-point binding (see below). The dissociation constant in the present model is given by

$$K_{\mathrm{d}} = \frac{1}{(n\delta V)} \left\{ \left[\exp\left(-\frac{E_{\mathrm{b}}^{\mathrm{P}}}{k_{\mathrm{B}}T}\right) + \exp\left(-\frac{E_{\mathrm{b}}^{\mathrm{A}'}}{k_{\mathrm{B}}T}\right) \right] \exp\left[-\frac{q_r q_l e^{-\alpha\langle r_1 \rangle}}{\varepsilon_{\mathrm{d}}\langle r_1 \rangle k_{\mathrm{B}}T}\right] + (n-1)\exp\left(-\frac{E_{\mathrm{b}}^{\mathrm{P}'} + E_{\mathrm{b}}^{\mathrm{A}}}{k_{\mathrm{B}}T}\right) \exp\left[-\frac{q_r q_l e^{-\alpha\langle r_2 \rangle}}{\varepsilon_{\mathrm{d}}\langle r_2 \rangle k_{\mathrm{B}}T}\right] \langle e^{\Delta S_2} \rangle \right\}^{-1},$$

[S3]

where δV is a volume of tolerance between the centers of mass of the two molecules in complex. As in Borg et al. (2007), the fractional Sic1 ligand bound

$$\theta = 1 + \frac{K_{\mathrm{d}}}{2\rho_l} - \left[\left(1 + \frac{K_{\mathrm{d}}}{2\rho_l}\right)^2 - 1\right]^{1/2},$$

[S4]

where ρ_l is total ligand concentration, $q_l = q_l^0 - 2n$, $q_l^0 = 11$, and the same α and δV values are used in the present analysis. Here we set the receptor charge to $q_r = 5$ (instead of $q_r = 3$ in Borg et al[5] to take into account the positive charges at both the **P** and **A** binding pockets.

Although the above development assumes that all CPDs are equivalent, our formulation can be further extended to accommodate CPDs with different binding properties. For example, eq.[S2] may be generalized to

$$Q_c^{(b)} \propto \sum_{i=1}^{n}\left[\exp\left(-\frac{E_{b,i}^{P}}{k_B T}\right)+\exp\left(-\frac{E_{b,i}^{A'}}{k_B T}\right)\right]\exp\left[-\frac{q_r q_l e^{-\alpha\langle r_1\rangle_i}}{\varepsilon_d\langle r_1\rangle_i k_B T}\right] + \sum_{i=1}^{n}\sum_{j=1,j\neq i}^{n}\exp\left(-\frac{E_{b,i}^{P'}+E_{b,j}^{A}}{k_B T}\right)\exp\left[-\frac{q_r q_l e^{-\alpha\langle r_2\rangle_{ij}}}{\varepsilon_d\langle r_2\rangle_{ij} k_B T}\right]e^{\Delta S_{ij}}\,,$$

[S5]

where the summations are over phosphorylated CPDs labeled by *i*,*j*. The contact energies, effective electrostatic distances, and the last conformational entropy term are now dependent upon the CPD(s) involved in the given quantity. The conformational entropy term may be estimated by the approximate relation $e^{\Delta S_{ij}} = \Omega(l_{ij};R)/\Omega_a^m$ defined in Song et al[7], where l_{ij} is the sequence separation between CPDs *i* and *j* and $R \approx 25$ Å is the spatial distance between the **P** and **A** binding pockets.

Since we are interested mainly in the general trend predicted by our model, we do not pursue this extended model further here. All numerical results presented in this study were for the simpler model described by eqs.[S2]—[S4] and computed using $\varepsilon_d = 20$ and a receptor to ligand concentration ratio $\gamma = 1$ as in Borg et al[5], with $\langle e^{\Delta S_2}\rangle = \Omega(\langle l_{ij}\rangle;R)/\Omega_a^m$ calculated using Ω quantities for a total chain length of 90 for Sic1 and an $\langle l_{ij}\rangle$ that corresponds to the chain length of twenty amino acid residues[7]. The results in Fig. 7c and Supplementary Fig. 7a were obtained by varying $\langle r_2\rangle$ while using the same set of contact energies, viz., $E_b^P = -10\ k_B T$, $E_b^{P'} = -5\ k_B T$, $E_b^A = -2\ k_B T$, and $E_b^{A'} = -3\ k_B T$. These contact energies satisfy the trend, suggested by experiment on pSic1, that binding is weaker for P′ than P, weaker for A′ than for P or P′, and weakest for A. The model is modified for the control computation in Supplementary Fig. 7b and c as well as the extensive scenario testing in Supplementary Fig. 7d. For the hypothetical scenario in Supplementary Fig. 7b, the physically reasonable contact energies quoted above did not produce an appreciable difference between models that allow **A**- pocket binding and those that permits only **P**- pocket binding. Thus lower physical contact energies were used to illustrate more extreme theoretical possibilities when non-contacting CPDs do not contribute to binding. For the other control computation in Supplementary Fig. 7c, an unphysical $q_l^0 = 0$ was used to illustrate an hypothetical situation with positive allostery in which two-point (P′,A) binding always dominates over single-point P or A′ binding.

To assess how the enhancement of ultrasensitivity in our physical picture depends on the choice of model parameters, we have considered 51×51=2,601 combined variations of the effective Sic1-Cdc4

polyelectrostatic interaction distance $\langle r_2 \rangle$ and contact energy $E_{\mathrm{P,A}} \equiv E_{\mathrm{b}}^{\mathrm{P}'} + E_{\mathrm{b}}^{\mathrm{A}}$ for two-point binding in our model while keeping $E_{\mathrm{b}}^{\mathrm{P}} = -10\ k_{\mathrm{B}}T$, $E_{\mathrm{b}}^{\mathrm{A}'} = -3\ k_{\mathrm{B}}T$, and $\langle r_1 \rangle$ = 12 Å constant. Ultrasensitivity (characterized by Δn) of these models as a function of the anticooperativity of the two Cdc4 pockets ($E_{\mathrm{P,A}}/|E_{\mathrm{P}} + E_{\mathrm{A}}|$) and the ratio $\langle r_2 \rangle/\langle r_1 \rangle$ of effective Sic1-Cdc4 electrostatic separation between one- and two-point binding (Supplementary Fig. 7d) exhibits compensatory influences from these two variables. Increasing ultrasensitivity (decreasing Δn) correlates with less anticooperativity (more negative $E_{\mathrm{P,A}}/|E_{\mathrm{P}} + E_{\mathrm{A}}|$) and with bound Sic1 conformations that are more “flattened” onto Cdc4 (smaller $\langle r_2 \rangle/\langle r_1 \rangle$). In the current modeling context, enhancement of ultrasensitivity is possible across a broad range of anticooperativity; but enhancement would require the two-point bound Sic1 conformational ensemble to be held spatially closer to Cdc4 (smaller $\langle r_2 \rangle/\langle r_1 \rangle$) when there is a high degree of anticooperativity (Supplementary Fig. 7d).

We note that while the results in Fig. 7c in main text were obtained using an electrostatics model, the trend predicted is general inasmuch as other mechanisms such as tethering[8] or transient kinetic trapping[9] can also lead to tighter Sic1-Cdc4 association and a steeper dependence of the transiently bound population on the number of phosphorylations when both **P** and **A** are engaged than when only **P** is engaged. This generalized physical picture may be represented by an approximate expression $K_{\mathrm{d}} \propto [A_1 \exp(c_1 n) + A_2 \exp(c_2 n)]^{-1}$ for the dissociation constant K_{d} where c_1 and c_2 characterize, respectively, the dependence of single- and two-point Sic1-Cdc4 binding on n ($c_2 > c_1 > 0$), and the factors A_1 and A_2 determine the minimum n at which **A** begins to contribute significantly. Thus the electrostatics model described by eq.[S3] corresponds to $c_1 = 2q_r e^{-\alpha\langle r_1 \rangle}/(\varepsilon_{\mathrm{d}}\langle r_1 \rangle k_{\mathrm{B}}T)$ and $c_2 = 2q_r e^{-\alpha\langle r_2 \rangle}/(\varepsilon_{\mathrm{d}}\langle r_2 \rangle k_{\mathrm{B}}T)$ in this generalized framework. If the allosteric pocket **A** contributes significantly only for intermediate and large n as in the extended polyelectrostatics model considered above, disabling **A** decreases the sharpness but does not affect the midpoint of ultrasensitive binding by much (Fig. 7c). However, if **A** always contributes significantly as long as there is more than one phosphorylated CPD, disabling **A** could lead to a significant shift in the midpoint of the ultrasensitive binding transition (Supplementary Fig. 7c).

Supplementary Note 4

Extension of the mathematical model to account for proximate states in pSic1-Cdc4 binding

As discussed in the main text, although our current model with three bound states and one unbound state provides a reasonable physical account of Sic1-Cdc4 binding, it does not fully explain the large negative allosteric effect observed in the Trp fluorescence binding experiments for the $Sic1^{20pS69/pS80}$ peptide. In addressing this issue, we first note that our mean-field treatment was designed specifically for the binding of full-length Sic1/$Sic1^{1-90}$ by considering a spatially extended ensemble of Sic1 conformations. As such it is not expected to perform well for relatively short Sic1 peptides. Nonetheless, our experimental observations of $K_a(\text{p69,p80}|\text{wt}) < K_a(\text{p69}|\text{wt}) + K_a(\text{p80}|\text{wt})$ and especially $K_a(\text{p69,p80}|\text{wt}) \approx K_a(\text{p69,p80}|\text{mt})/2 < K_a(\text{p69,p80}|\text{mt})$ posed a conceptual challenge to our assumption of having a single Sic1 unbound state. It is clear that there are more bound $Sic1^{20pS69/pS80}$ configurations for wild-type than for mutant Cdc4 because the former has two binding pockets whereas the latter has only one (Fig. 4c). If there is only one unbound state, how can the wild-type Cdc4, when compared to mutant Cdc4, have less bound population relative to the unbound population?

This consideration together with what we have learned about the negative allostery between the two Cdc4 binding pockets in this work led us to hypothesize the existence of *proximate* bound and unbound states in Sic1-Cdc4 association (Supplementary Fig. 7e). These states have one (for bound Sic1) or two (for unbound Sic1) phosphate(s) maintained near one or both of the Cdc4 binding pockets, probably by electrostatic attraction, but without direct contact between the phosphates and the Cdc4 pockets. It is intuitive to envision that these states can be induced kinetically, and thus favored thermodynamically, by the process of frequent binding-release-rebinding induced by the negative allostery between the two Cdc4 pockets (Fig. 7a). While significant future effort will be needed for developing a more intricate mathematical formulation for this expanded view of the Sic1-Cdc4 system, it is useful to explore how the concept of proximate states can qualitatively rationalize our experimental data here.

With the contributions from proximate states, there would be a total of five bound states (all three in Fig. 7b and the two in Supplementary Fig. 7e ii, iii). Because the Sic1 conformations are similarly constrained in the proximate bound states in Supplementary Fig. 7e as in the two-point bound state in Fig. 7b, their effective electrostatic separations with Cdc4 is expected to be similar, viz., all approximately equal to an $\langle r_2 \rangle$ that is smaller than the $\langle r_1 \rangle$ for the two one-point bound states in Fig. 7b. In this scenario, since the Sic1 conformational ensembles of the proximate bound states are held closer to the Cdc4 and thus allowing for stronger long-range electrostatic interactions, they can play a role that is mathematically equivalent to that of the two-point bound state to enhance Sic1-Cdc4

binding ultrasensitivity. Consequently, the prediction of enhanced ultrasensitivity by the current model should apply to the expanded model as well. Moreover, with enhanced ultrasensitivity relying partially on proximate bound states and not entirely on the two-point bound state, enhanced ultrasensitivity in the expanded scenario can be effectuated in the presence of even stronger anticooperativity between the two Cdc4 pockets than that assumed in the current model. In other words, the predicted feature of enhanced ultrasensitivity is expected to be even more robust in an expanded model that considers proximate states than in the current model.

Proximate states can provide rationalization for the otherwise puzzling $Sic1^{20pS69/pS80}$ binding data as well. The existence of a proximate yet unbound state of $Sic1^{20pS69/pS80}$ for wild-type Cdc4 (Supplementary Fig. 7f v) explains $K_a(p69,p80|wt) < K_a(p69|wt) + K_a(p80|wt)$ because this proximate unbound state, which increases the total unbound population and therefore decreases K_a, is present for $Sic1^{20pS69/pS80}$ but not for $Sic1^{20p69}$ or $Sic1^{20p80}$. The proximate bound states of $Sic1^{20pS69/pS80}$ of wild-type Cdc4 (Supplementary Fig. 7f iii, iv) offer a plausible explanation for $K_a(p69,p80|wt) \approx K_a(p69,p80|mt)/2 < K_a(p69,p80|mt)$ if these configurations together with the unbound proximate state dominate over configurations in which the unbound phosphate in the Sic1 peptide is more free to position itself to achieve a stronger long-range attractive electrostatic interaction with the primary Cdc4 pocket, as is the case for mutant Cdc4 where the allosteric pocket is disabled (Supplementary Fig. 7f i). A similar effect should also be present for full-length Sic1 or $Sic1^{1\text{-}90}$ binding. In that case, however, as long as the number of phosphorylations $n > 3$, there are phosphates (one phosphate for $n = 3$) that can take part in long-range electrostatic interactions with the primary Cdc4 pocket even if one of the Sic1 phosphates is tied down in the vicinity of the allosteric pocket. Hence the corresponding anti-cooperative effect is expected to be less drastic than that exhibited by $Sic1^{20pS69/pS80}$ binding. Although quantitative details remain to be developed, this line of reasoning should allow for accommodation of our experimental result $K_a(pSic1^{1\text{-}90}|wt) \approx 4K_a(pSic1^{1\text{-}90}|mt) > K_a(pSic1^{1\text{-}90}|mt)$ (Table 2) in an expanded model that considers proximate states, such that the expanded model can predict a similar trend for $Sic1/Sic1^{1\text{-}90}$ binding that has already been captured in our current mathematical model.

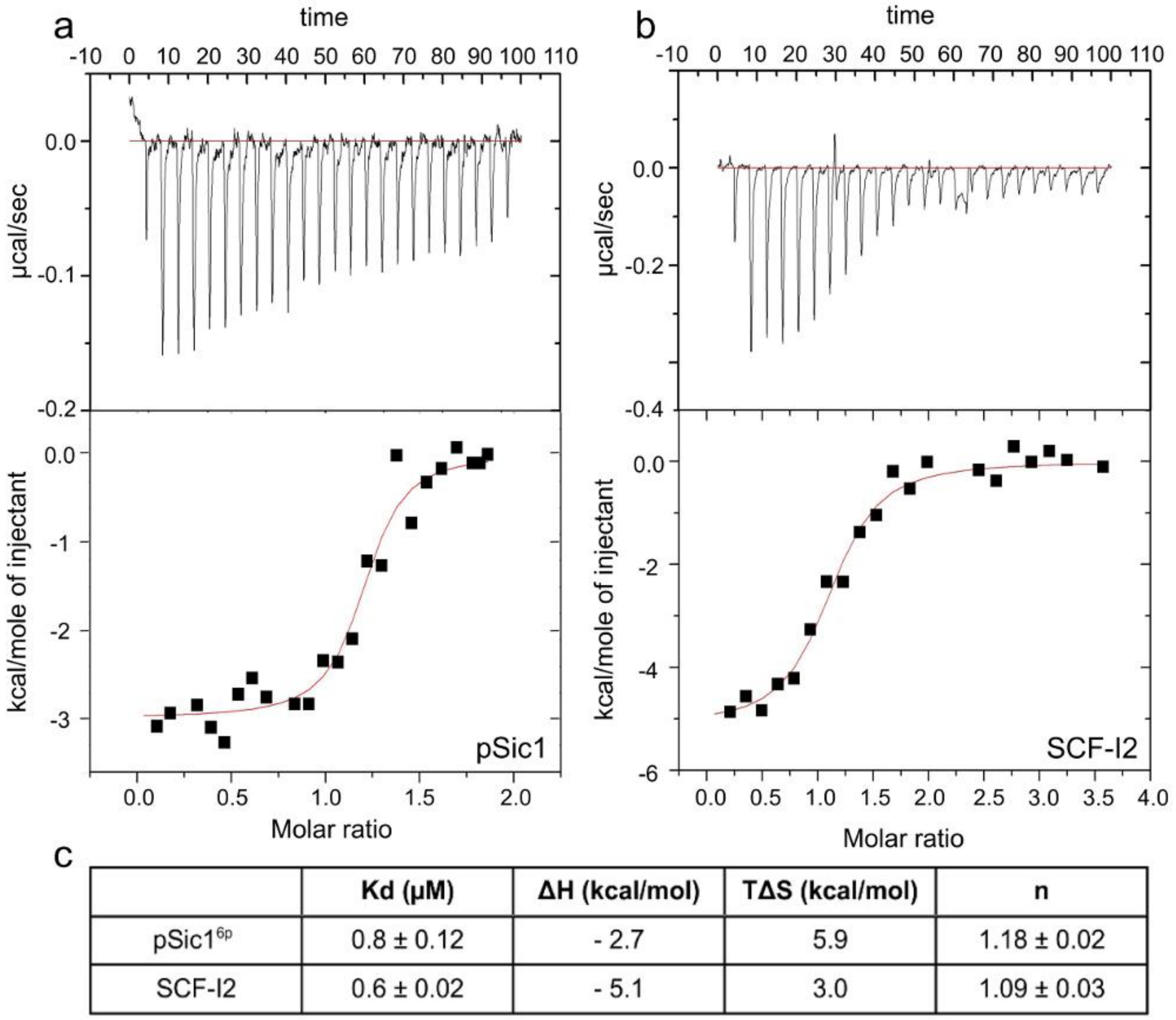

c

	Kd (µM)	ΔH (kcal/mol)	TΔS (kcal/mol)	n
pSic1[6p]	0.8 ± 0.12	- 2.7	5.9	1.18 ± 0.02
SCF-I2	0.6 ± 0.02	- 5.1	3.0	1.09 ± 0.03

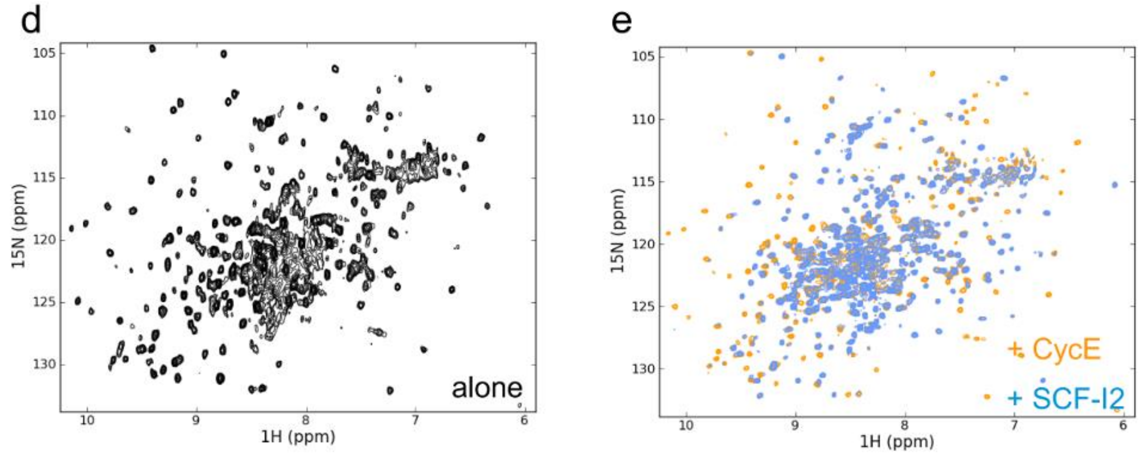

Supplementary Figure 1. Characterization of isolated Cdc4^{WD40} domain.

(**a-b**) Isothermal titration calorimetry (ITC) data for binding of the Cdc4^{WD40} to (**a**) pSic1 and (**b**) SCF-I2. **(c)** Thermodynamic parameters for the interaction of Cdc4^{WD40} and pSic1 and SCF-I2. (**d-e**) ^{1}H-^{15}N HSQC spectra of the ^{15}N labelled Cdc4WD40 (**d**) in the absence and (**e**) in the presence of CycE peptide (orange) or SCF-I2 (blue).

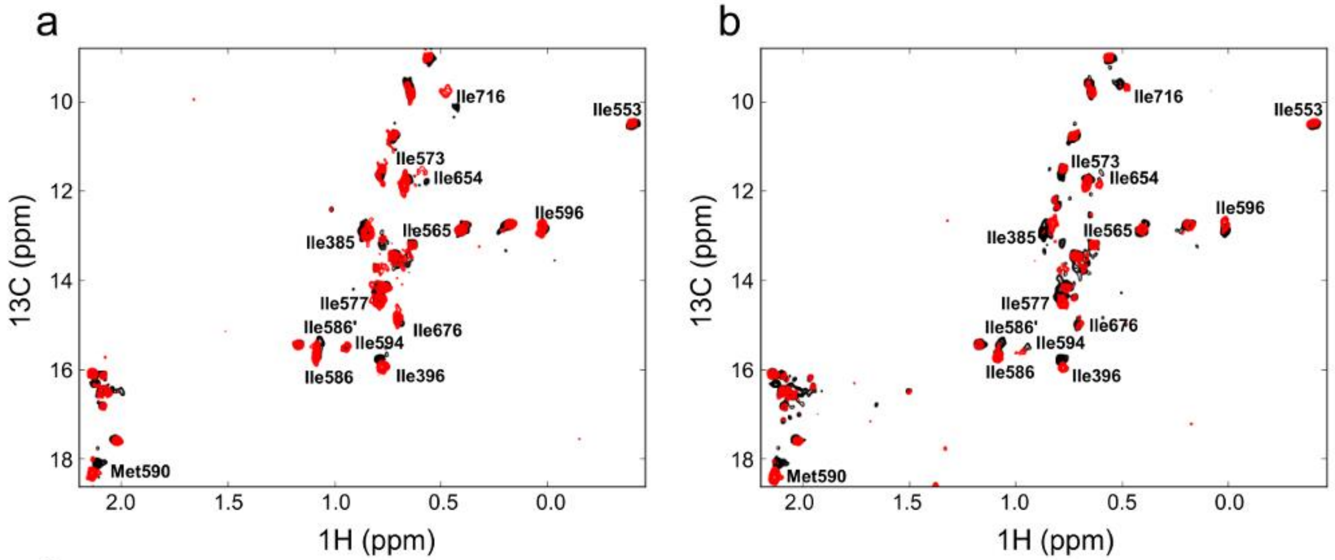

c

Cdc4 phospho-degron: CPD

L/I – L/I/P – pT – P – <RK>$_4$ <> disfavored residues

Sic1							
T2:		Nt–	M	pT	P	S	T	P
T5:	T	P	S	pT	P	P	R	S
T33:	G	Q	K	pT	P	Q	K	P
T45:	V	P	V	pT	P	S	T	T
S69:	G	M	T	pS	P	F	N	G
S76:	G	L	T	pS	P	Q	R	S
S80:	P	Q	R	pS	P	F	P	K
T173:	V	P	G	pT	P	S	D	K
S191:	N	N	N	pS	P	K	N	D
CycE peptide								
		L	L	pT	P	P	Q	S
Ash1 peptides								
pThr286	W	S	I	pT	P	P	V	T
pThr290	P	P	V	pT	P	P	M	S
pSer294	P	P	M	pS	P	P	T	N

Supplementary Figure 2. Substrate recognition by Cdc4^{WD40} domain.

(**a**) ^{1}H-^{13}C TROSY HMQC spectra of the IM-labelled Cdc4^{WD40} in the absence (black) and in the presence of CycE (red). (**b**) ^{1}H-^{13}C TROSY HMQC spectra of the IM-labelled Cdc4^{WD40} in the absence (black) and in the presence of pSic1 (red). (**c**) Consensus sequence of Cdc4 phosphodegron (CPD) and sequences of the different CPD sites of Sic1, CycE and Ash1 substrates from P-3 to P+4 positions. The residues in P-2, P-1 and P-3 positions, which showed different engagements in our studies, are highlighted in grey.

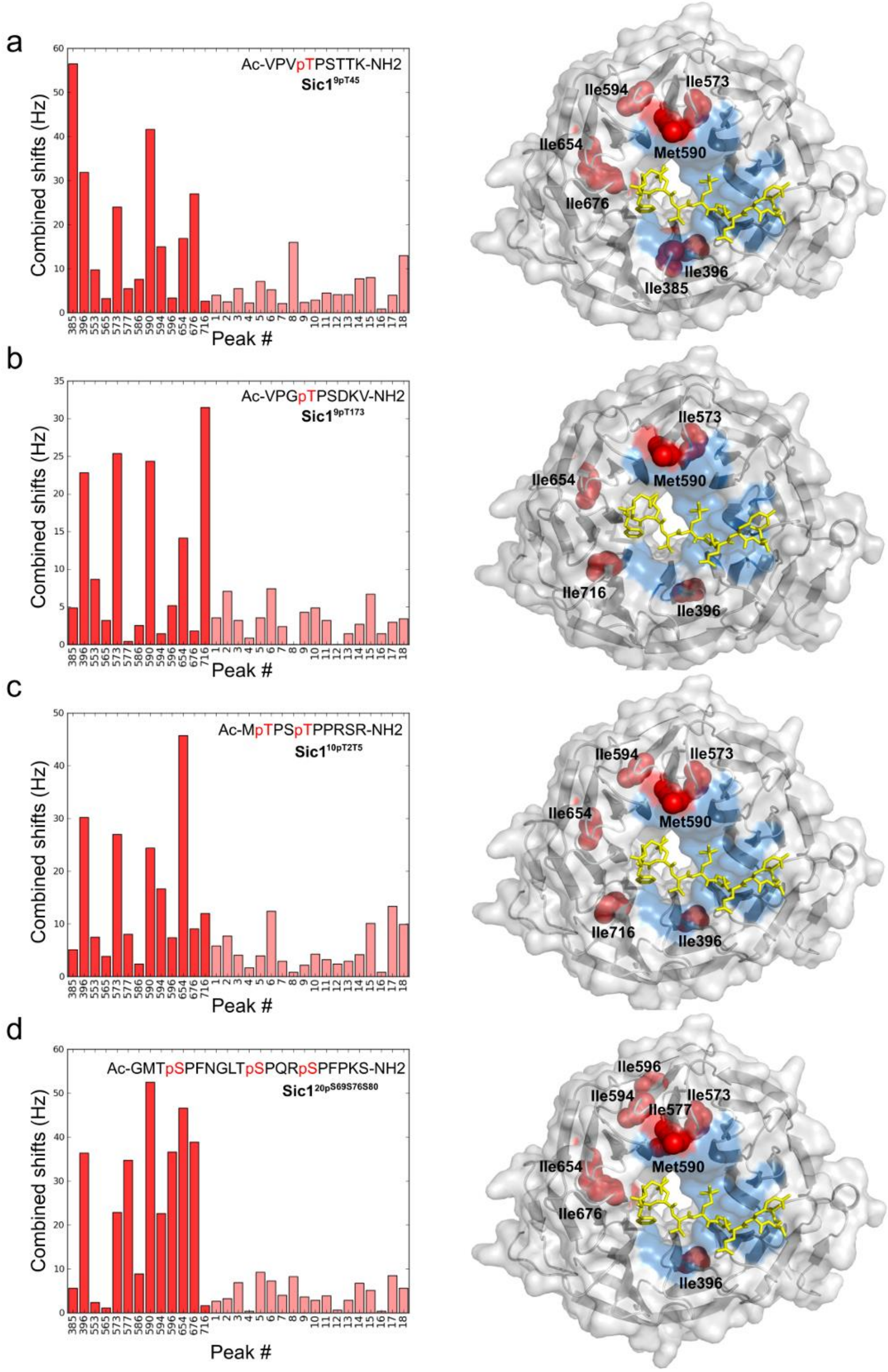

Supplementary Figure 3. Binding of pSic1 phosphopeptides to Cdc4^{WD40} domain.

(**a-d**) Combined ^{1}H and ^{13}C chemical shifts of Cdc4^{WD40} upon binding (**a**) Sic1^{9pT45}, (**b**) Sic1^{9pT173}, (**c**) Sic1$^{10pT2/pT5}$ and (**d**) Sic1$^{20pS69/pS76/pS80}$, with chemical shifts mapped on the structure of Cdc4^{WD40} (3V7D). Assigned Ile and Met residues are in red, labeled with the residue number, un-assigned residues in pink, Sic1 peptide in yellow, CPD binding pocket from[2] in blue and residues showing significant chemical shift changes in red spheres.

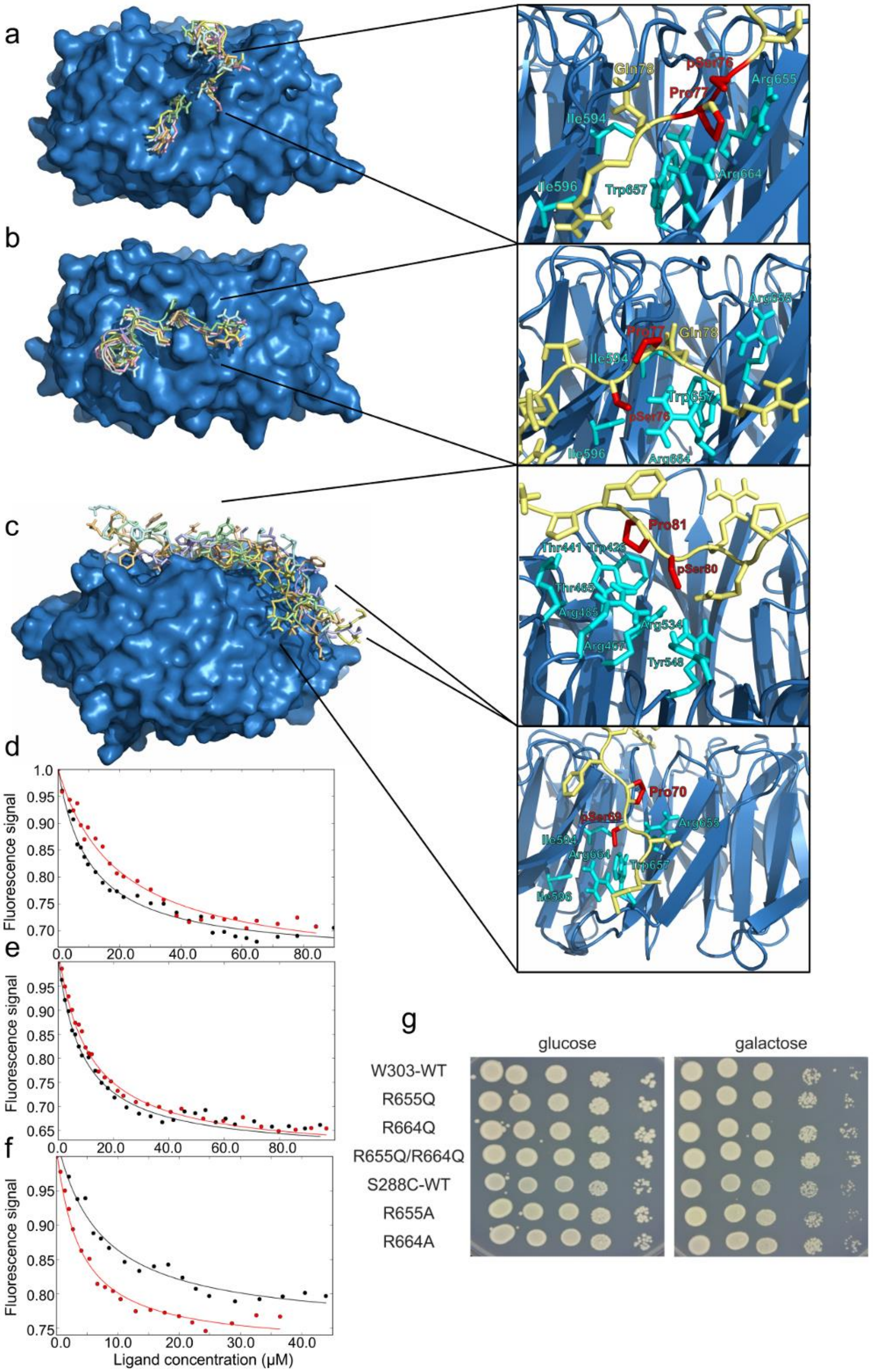

Supplementary Figure 4. Characterization of the allosteric binding pocket on Cdc4^{WD40}.

(**a-b**) Ensemble of 10 lowest-energy HADDOCK structures for Cdc4^{WD40} with the Sic1$^{20pS69/pS76/pS80}$ peptide in the allosteric pocket for sets of conformations with (**a**) potential contacts of another CPD site with the primary binding pocket and (**b**) incompatible for binding of another CPD site to the primary pocket. (**c**) Ensemble of 10 lowest-energy HADDOCK structures for Cdc4^{WD40} with the Sic1^{20pS69pS80} peptide bound simultaneously at the primary and the allosteric pocket. The Cdc4^{WD40} domain is shown

in blue surface and peptides in multiple binding modes in variously colored stick representation. Docking in the allosteric pocket (**a-c**) and in the primary pocket (**c**) is enlarged, with critical contact residues shown in stick representations for $Cdc4^{WD40}$ (blue) and peptide (yellow). (**d-e**) Trp fluorescence binding curves for interactions of (**d**) $Sic1^{20pS69}$, (**e**) $Sic1^{20pS80}$ and (**f**) $Sic1^{20pS69pS80}$ peptides with wild-type (black) and mutant (R664Q) $Cdc4^{WD40}$ (red). (**g**) Colony growth phenotype of yeast strains bearing the indicated *CDC4* alleles. The indicated strains were spotted on selective glucose or galactose medium at 30°C for 3 days to assess growth in the absence and presence of *GAL1-SIC1* induction, respectively.

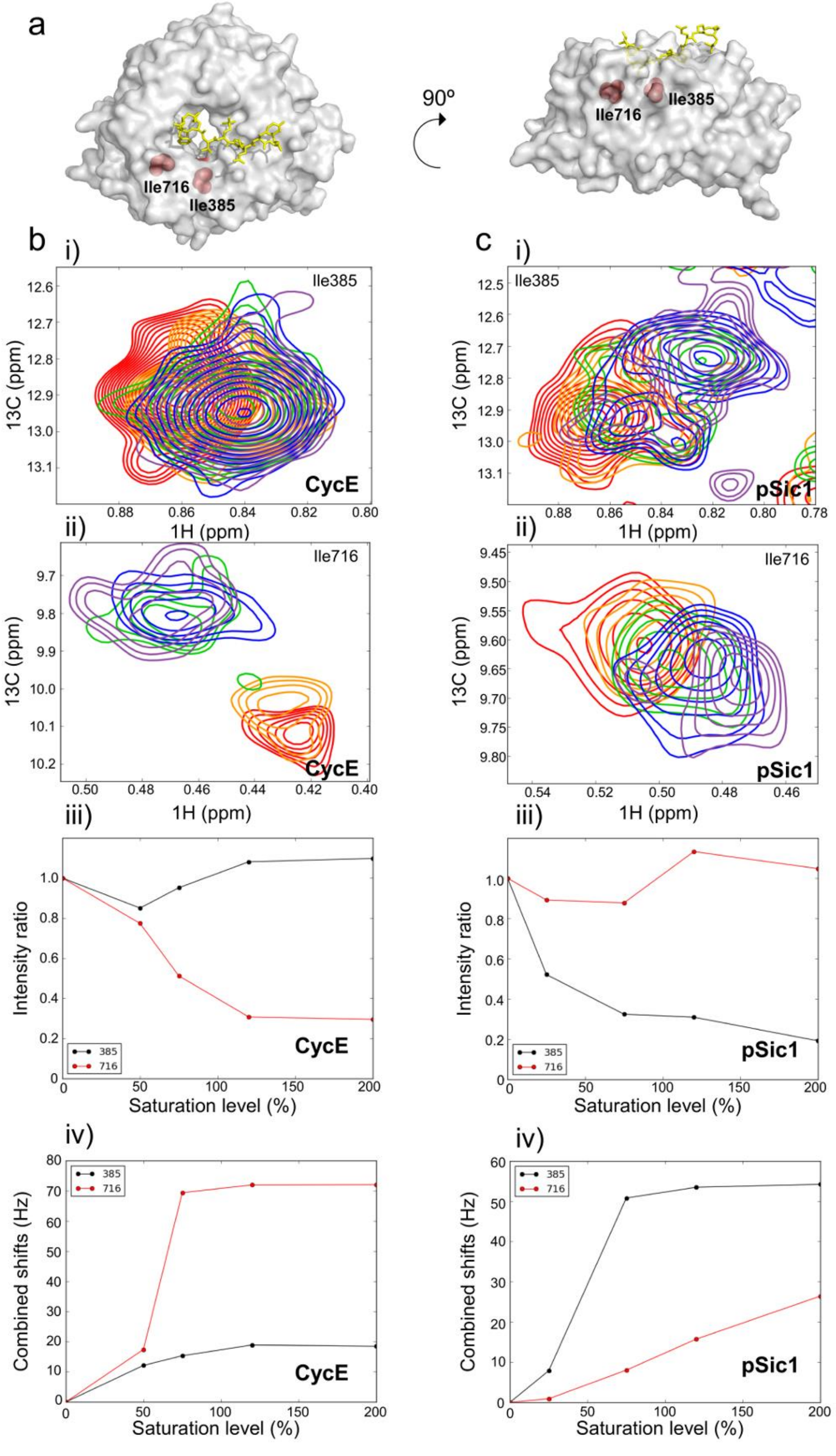

Supplementary Figure 5. NMR perturbations probing the P-1 position.

(**a**) Surface representation of Cdc4^{WD40} with Sic1 peptide shown in yellow. The residues in the P-1 pocket are displayed in red spheres. (**b-c**) ^{1}H-^{13}C TROSY HMQC spectra of residues (i) Ile385 and (ii) Ile716 in the absence (red) and presence of increasing amounts (50% (orange), 75% (green), 120% (blue), 200% (purple) of (**b**) CycE peptide or (**c**) pSic1, (iii) intensity ratios of the peaks corresponding to the unbound states and (iv) chemical shifts of the peaks corresponding to the bound states at different saturation levels.

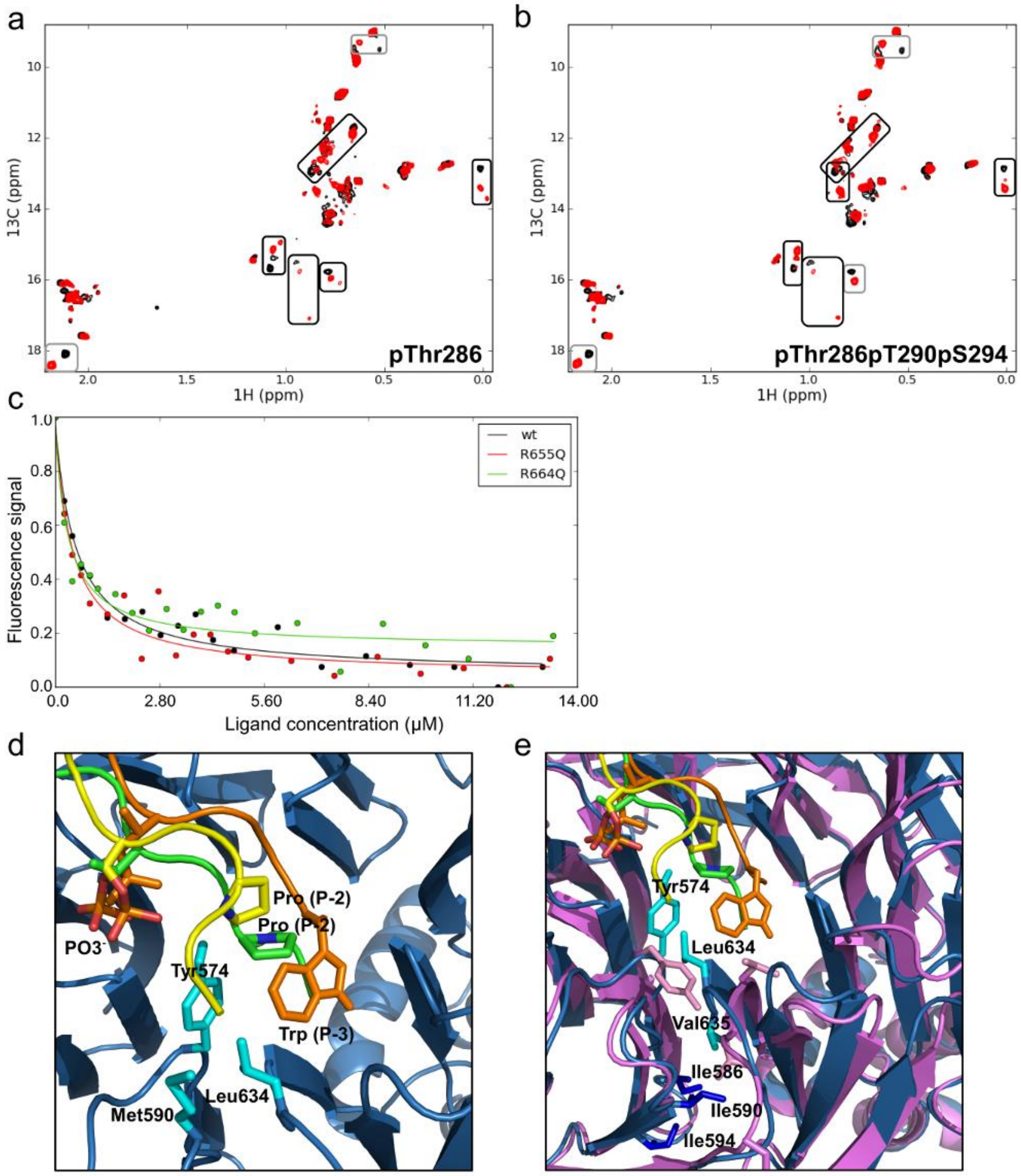

Supplementary Figure 6. Interactions of Ash1 motifs with Cdc4^{WD40}.

(**a-b**) ^{1}H-^{13}C TROSY HMQC spectra of the IM-labelled Cdc4^{WD40} in the absence (black) and presence of (**a**) pThr286 Ash1 peptide or (**b**) triply phosphorylated Ash1 peptide (red). Black rectangles indicate residues with two different bound states; grey rectangles indicate residues with one bound state. (**c**) Trp fluorescence binding curves for interactions of triply phosphorylated Ash1 peptide with wild-type, R664Q and R655Q Cdc4^{WD40}. (**d**) The lowest-energy HADDOCK structures for Cdc4^{WD40} complexes with pT286 Ash1 (orange), pT290 Ash1 (green), and pS294 Ash1 (yellow) peptides. Phosphorylated residues and residues in P-2 positions for pT290 and pS294 and the residue in P-3 position for pT286 are shown in stick representation. Cdc4 residues lining the P-2 pocket are in blue stick representation. (**e**) Overlay of the HADDOCK structures for Ash1-Cdc4 complexes (blue) and SCF-I2 bound Cdc4-Skp1 X-ray structure (3MKS, magenta). Cdc4 residues lining the P-2 pocket which differ between the Ash1 complexes (cyan) and SCF-I2 complex (magenta) are shown in stick representation. Isoleucines showing chemical shift changes upon SCF-I2 binding are shown in blue stick representation.

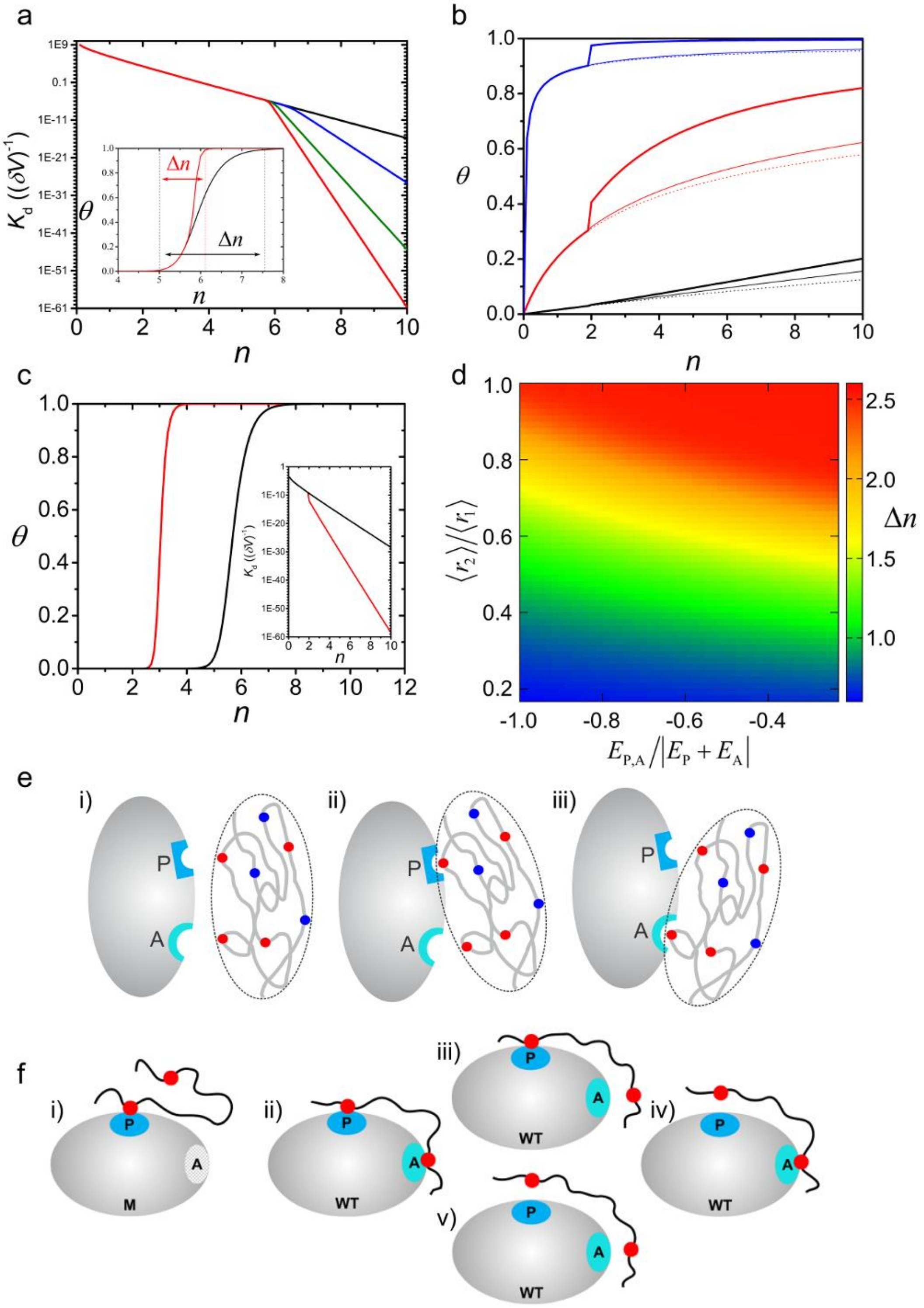

Supplementary Figure 7. A biophysical model for dynamic site exchange.

(**a**) Dissociation constants K_{d} versus *n* (number of phosphorylation sites) for the four models in Fig. 7c of the main text are shown using the same coloring. As noted in supplementary note 3, $q_r = 5$, $q_l^0 = 11$. In units of $k_{\mathrm{B}}T$, $E_{\mathrm{b}}^{\mathrm{P}} = -10$, $\left(E_{\mathrm{b}}^{\mathrm{P}'}, E_{\mathrm{b}}^{\mathrm{A}}\right)$, $E_{\mathrm{b}}^{\mathrm{A}'} =$ (−5,−2),−3, and $\rho_l = 10^{-6}(\delta V)^{-1}$ were used for these models, which exhibit dominance of the two-point (P′,A) binding over single-point P or A′ binding for $n \gtrsim 6$. Inset: Ultrasensitivity is quantified by the transition width Δ*n*, defined here as Δ*n* =

$n(\theta = 0.99) - n(\theta = 0.01)$. Two transition curves from Fig. 7c and their Δn spans are shown, as examples, where $\Delta n = 2.53$ for the model that admits only **P**-pocket binding (black curve). (**b**) Binding curves for *hypothetical* cases in which the phosphorylated CPDs that are not directly engaged with the Cdc4 binding pockets do not contribute to binding. This situation is mathematically equivalent to setting $q_r = 0$ in eqs. [S2] and [S3] of our model. Results from models with the same contact energy E_b^P for the **P** pocket are shown with the same coloring. In units of $k_B T$, $E_b^P = -12$ (black), -15 (red), and -20 (blue). Solid curves are for models in which both the **P** and **A** pockets can engage whereas dotted curves are for models in which only the **P** pocket can engage. The dotted curves (bottom among each set of curves in the same color) are identical to those shown in Fig. 2b of (Borg et al., 2007). The contact energies for the models represented by the thin solid curves are $(E_b^{P'}, E_b^A)$, $E_b^{A'} = (-11,-4), -6$ (black); $(-14,-4),-6$ (red); and $(-19,-4),-6$ (blue). Those represented by the thick solid curves are $(E_b^{P'}, E_b^A)$, $E_b^{A'} = (-11,-5), -8$ (black); $(-14,-7),-10$ (red); and $(-19,-10),-15$ (blue). As in Fig. 2b of Borg et al.[5], $\rho_l = 10^{-7}(\delta V)^{-1}$ for all the models in this panel. The θ versus n relations here indicate that certain choices of contact energies that allow for two-point (P′,A) binding that simultaneously engages both the **P** and **A** pockets to dominate can lead to steeper slopes for $n \geq 2$ but none of the hypothetical binding curves is sigmoidal. (**c**) Binding curve and the corresponding K_d versus n curves (*inset*) for a hypothetical model with positive allostery in which two-point (P′,A) binding dominates over single-point P or A′ binding for $n \geq 2$. The present example was obtained by setting $q_l^0 = 0$ for the $\langle r_2 \rangle$ = 8 Å model in (**c**) and $\rho_l = 10^{-18}(\delta V)^{-1}$ while other parameters remain unchanged. Comparing this model (red) with the corresponding model in which **A**-pocket binding is disabled (black) indicates, in this *hypothetical* scenario, that the midpoint n value for ultrasensitive transition would shift from a smaller to a large n upon disabling binding at the **A** pocket. (**d**) Variation of ultrasensitivity measure Δn (see Inset of **a**) in our model is presented as a heat map (color code on the right) as a function of the effective polyelectrostatic distance ratio $\langle r_2 \rangle / \langle r_1 \rangle$ and the degree of negative allostery (anticooperativity) between the two Cdc4 binding pocket characterized by $E_{P,A} / |E_P + E_A|$, where $E_{P,A} \equiv E_b^{P'} + E_b^A$, and $|E_P + E_A|$ is the absolute value of $E_b^P + E_b^{A'}$. The additive case (no anticooperativity) corresponds to $E_{P,A} / |E_P + E_A| = -1$, whereas a less negative value of $E_{P,A} / |E_P + E_A|$ means a higher degree of anticooperativity. (**e**) Possible proximate states of Sic1 or Sic1[1-90] (same schematic representation as in Fig. 7b, viz., chain in dashed envelope, negatively charged phosphorylated CPDs and positively charged residues are represented, respectively, by red and blue circles) induced by anticooperativity between the P and A pockets in wild-type Cdc4 (solid ellipse). (i) A proximate unbound state with two phosphates each in the vicinity of one of the two Cdc4 binding pockets. (ii, iii) Proximate bound states in which one phosphate binds to (is in contact with) one of the two binding pockets while the other phosphate is in close proximity to but not in contact with the other Cdc4 pocket. (**f**) (i, ii) Simple bound states and (iii, iv) possible proximate bound and (v) unbound states of Sic1[20pS69/pS80] peptide (black chain with the phosphates represented by red circles) in association with (i) mutant and (ii-v) wild-type Cdc4.

References

1. Mittag, T. et al. Dynamic equilibrium engagement of a polyvalent ligand with a single-site receptor. *Proc Natl Acad Sci U S A* **105**, 17772-7 (2008).
2. Orlicky, S., Tang, X., Willems, A., Tyers, M. & Sicheri, F. Structural basis for phosphodependent substrate selection and orientation by the SCFCdc4 ubiquitin ligase. *Cell* **112**, 243-56 (2003).
3. Orlicky, S. et al. An allosteric inhibitor of substrate recognition by the SCF(Cdc4) ubiquitin ligase. *Nat Biotechnol* **28**, 733-7 (2010).
4. Dominguez, C., Boelens, R. & Bonvin, A.M. HADDOCK: a protein-protein docking approach based on biochemical or biophysical information. *J Am Chem Soc* **125**, 1731-7 (2003).
5. Borg, M. et al. Polyelectrostatic interactions of disordered ligands suggest a physical basis for ultrasensitivity. *Proc Natl Acad Sci U S A* **104**, 9650-5 (2007).
6. Zhou, H.X. Quantitative account of the enhanced affinity of two linked scFvs specific for different epitopes on the same antigen. *J Mol Biol* **329**, 1-8 (2003).
7. Song, J., Ng, S.C., Tompa, P., Lee, K.A.W. & Chan, H.S. Polycation-pi interactions are a driving force for molecular recognition by an intrinsically disordered oncoprotein family. *PLoS Comput Biol* **9**, e1003239 (2013).
8. Deshaies, R.J. & Ferrell, J.E., Jr. Multisite phosphorylation and the countdown to S phase. *Cell* **107**, 819-22 (2001).
9. Klein, P., Pawson, T. & Tyers, M. Mathematical modeling suggests cooperative interactions between a disordered polyvalent ligand and a single receptor site. *Curr Biol* **13**, 1669-78 (2003).
